# Cervical Cancer, Different Treatments and Importance of Bile Acids as Therapeutic Agents in This Disease

**DOI:** 10.3389/fphar.2019.00484

**Published:** 2019-06-04

**Authors:** Tanja Šarenac, Momir Mikov

**Affiliations:** Department of Pharmacology, Toxicology and Clinical Pharmacology, Faculty of Medicine, University of Novi Sad, Novi Sad, Serbia

**Keywords:** bile acids, cervical cancer, different treatments, therapeutic agents, treatment of cervical cancer

## Abstract

Cervical cancer can be cured, because it has a long preinvasive period. Early diagnosis and treatment of cervical cancer at women are crucial for reducing of rate mortality. Today, there are many methods for detecting premalignant lesions and one of them is a conventional Papanicolaou test. Cervical cancer develops through a series of changes in the epithelium called cervical intraepithelial neoplasia (CIN). The biological and genetic characteristics of the cells at cancer *in situ* are irreversibly altered and abnormal cells have the potential to metastasize to others anatomical regions. Infection with human Papillomavirus, which is transmitted sexually, is considered the main cause and represent the necessary, but not the only factor for the development of cervical cancer. Types of high risk human Papillomavirus are often associated with invasive cervical cancer. The carcinogenic types of HPV 16 and 18 are responsible for 70% of cervical cancer and about 50% of CIN 3. Primary prevention of cervical cancer is aimed at reducing incidence, control of causes and risk factors. In this scientific work, in addition to explaining the various treatments necessary for the treatment of cervical carcinoma, we were discussed about the anticancer effects of the synthetic derivative of ursodeoxycholic acid, such as HS-1183, and synthetic derivatives of chenodeoxycholic acid such as HS-1199 and HS-1200. Also, the effects of bile acid complexes with metals such as platinum, zinc, nickel, and copper were considered in the effective treatment of cervical cancer.

KEY POINTS

• Lymphogenic spreading of cervical cancer occurs relatively early in the regional lymph nodes, while this sort of progression of cervical cancer is rarer in the juxtaregional (paraaortic), mediastinal and supraclavicular nodes.

Clinically proven supraclavicular metastases are not a rarity. In stages IIb and IIIa with metastases in paraaortal nodes occur a 20% metastases at the neck lymph nodes.

Hematogenic metastases are relatively rare and occur in the posterior phase. Distant metastases are detected in the lungs and liver.

Preinvasive and microinvasive stages of cervical cancer are without symptoms. With deeper invasion of the strome, certain clinical symptoms such as prolonged menstruation, increased vaginal secretions, vaginal bleeding between the two periods, contact bleeding (after coitus), unilateral pelvic pain with spreading in hip joint (infiltration of the pelvic nerve plexus), dysuric disturbance, anemia, islet of the lower extremities.

In order to diagnose the level spreading of primary lesion of cervical cancer most commonly are used the supplemental searches such as cytoscopy, rectoscopy, urography, irigography, lung and bone radiography, scintigraphy of the liver, kidney and bone, lymphography, CT (MR) of abdomen and pelvis, as well as laboratory analysis.

Surgical treatment consists of transvaginal hysterectomy, transabdominal removal of the uterus (via laparotomy), bilateral adenectomy (removal of the ovaries and the fallopian tubes), upper and middle third of the vagina and lymphonodectomy of the regional lymph nodes. The most commonly used radiotherapy, intracavitary brachytherapy, manual afterloading technique and remote afterloading techniques.

The synthetic derivatives of ursodeoxycholic acid and chenodeoxycholic acid such as HS-1183, HS-1199, and HS-1200 are used to treat cervical cancer. These derivatives of chenodeoxycholic acid and ursodeoxycholic acid are capable of inhibiting cell proliferation and inducing apoptosis in SiHa human cells of cervix.

Platinum compounds are used as catalysts in cervical cancer therapy. Clinical use of platinum complexes for which the bile acids bind is based on the desire to achieve the death of tumor cells and the spectrum of drug activity in the treatment of cervical cancer.

Bisursodeoxycholate (ethylenediamine) platinum (II) [Pt(UDC)_2_(en)] is characterized by important cytotoxicity against HeLa cervical carcinoma cells and this effect already being clearly detectable after 24 h.

## Introduction

Malignant cervical tumors are one of the most common malignancies in the female population. The incidence ranges from 8 to 30 newly detected cases per 100,000 women per year, depending on the country and region (Denny, 2012). In the clinical material of the Institute of Oncology and Radiology in Belgrade, these tumors constitute approximately 50% of all malignancies of the reproductive system of women. Most often, these tumors are discovered in the 5th and 6th decades of life. They rarely occur in people under the age of 20 years. Many factors have been found to influence the malignancy to a greater or lesser extent: early coitus (before the age of twenty), promiscuity (multiple sexual partners, pregnancy in younger age, higher number of births, viral infection (HSV-herpes simplex virus), HPV (Papillomavirus), poor socio-economic status of women, and others (Dillner et al., 2008). On the other hand, it has been observed that the incidence of this malignancy is lower among people who practice circumcision. This moment is associated with hygiene of reproductive organs (Jensen et al., 2004). Relatively low incidence of this malignancy in Jewish women is explained by the fact that in this nation there is a circumcision practice, that women begin relatively late sexual relations, they are getting married late and they have a relatively small number of births (Jensen et al., 2004). Most researches think that heredity does not play any role in the development of cervical cancer. Cervical cancer develops either from the back lip, from the central canal or from the front lip of the cervix. The main three forms of growth of this tumor are: ulcerative, exophytic, endophytic.

**FIGURE 1:**
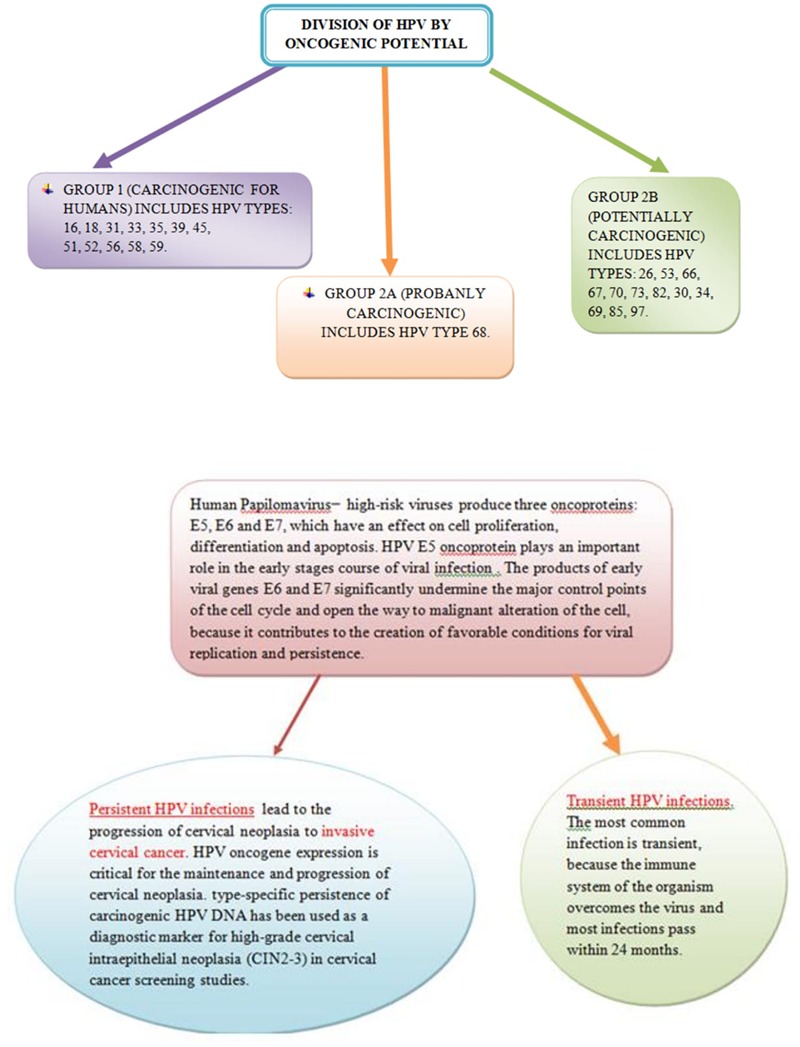
Persistent Human papillomavirus (HPV) infection leads to the invasive cervical cancer and transient HPV infections pass within 24 months.

Ulcerative lesion on the cervix is revealed as excavation ulceration and with central necrosis and irregular, hard and infiltrated edges. The exophytic lesion has the appearance of a cluster tumor similar to cauliflower, which is friable and soft and which spontaneously or contact bleed. Necrotic changes of tumor are typical (Dillner et al., 2008). A massive exophytic form (bulky form) destroys the edges of the cervix and dilates the vagina. Diameter of cervical cancer is still higher than 4 cm. An endophytic form (endocervical growth) gives the appearance of the cervix like barrel, which is late revealing and it is very insidious. Mixed exophytic-ulcerative forms also occur in clinical practice. Planocellular (squamous cell carcinoma) is the most common pathohistological form of cervical tumor and represents over 90% of all cancers occurring at this site (Anttila et al., 2001). Approximately 6% of cervical tumors are adenocarcinomas and less than 2% are adenosquamous (mixed) carcinomas (Katanyoo et al., 2012). Also, there are rare carcinomas and cervical sarcomas (Janicek and Averette, 2001; Chankapa et al., 2011). According to the degree of differentiation, cervical cancers are divided into: g_x_, g_1_, g_2_, g_3_, g_4_ (unknown, good, medium, poor and undifferentiated grades). Cervical carcinoma spreads directly (“per continuitatem”) to the cervical channel, the uterine body, vaginal vestibule, vagina, parametrium, urinary bladder and rectum. Lymphogenic spread of cervical cancer occurs relatively early and often in regional lymph nodes, while this spread is rarely in juxtaregional (paraaortal) mediastinal and supraclavicular nodes (Schwartz et al., 2009). In microinvasive cervical cancer lymphogenic spread with invasion of strome to 3 mm in depth almost does not exist (less than 1%), and with stromal invasion from 3 to 5 mm this spread is rare (5–7%). In the stage Ib (FIGO), 15–20% of patients have pelvic (regional) metastases, then in stage IIb about 35%, and in stage III about 60% (Sankaranarayanan, 2006; Zhilan et al., 2016). Paraaortic nodes were attacked in less than 2% of cases in the IIb–III stage of the disease. Clinically proven supraclavicular metastases are not a rarity. At stage IIb–III with metastases in paraaortal nodes, about 20% of metastases at the neck lymph nodes are detected. Hematogenic metastases are relatively rare and occur quite late, so the cervical cancer is a pelvic disease for a long time (Xiaotian et al., 2017). Most common metastases are found in the lungs and liver. Bone metastases are rarely detected and most commonly found in the vertebrae of the spinal column and long bones of the lower extremities (rarely). Brain metastases are also rare as in the kidneys, adrenal glands, colon and pancreas. The frequency of distant metastases increases with increasing local spread of the disease. If invasive carcinoma of cervix is not treated the lethal outcome occurs in all cases, within the first 3 years of the onset of the disease. The most common of death is renal insufficiency with urea due to bilateral obstruction of the ureter, then hemorrhage, ileus and metastases in the lungs and liver. Preinvasive (zero stage) and microinvasive (stage Ia) cervical carcinoma are without symptoms (Koh et al., 2013). Only with deeper stromal invasion, cervical cancer causes certain clinical symptoms and signs: prolonged menstruation, increased vaginal secretions, vaginal bleeding between the two periods, contact bleeding (after coitus), unilateral pelvic pain with propagation in the joint of the hip (infiltration) of the pelvic plexus), dysuria, constipation, diarrhea, anemia and the swelling of lower extremity. Clinical (gynecological) examination consists of vaginal, rectal and abdominal palpation, vaginal inspection by speculum (ecarter) and colposcopy. For the detection of preinvasive (Ca *in situ*) and microinvasive cervical carcinoma (the depth of stromal invasion less than 5 mm), Schûller’s test (coloring of the cervix surface with Lugol solution) is used, cytological analysis of the vaginal secretion by Papanicolaou, and colposcopy (Antic et al., 2012). The most commonly attacked regional lymph nodes are external iliac, then obturatorial and internal iliac. Invasive cervical cancer is clinically clearly noticed by inspection and palpation (Antic et al., 2012). It detects a lesion on the cervix in the form of ulceration, infiltration or exophytic growth, which it has expanded to a greater or lesser extent on the surrounding anatomical structures. Biopsy from the edge of the lesion and histopathological analysis of the material finally give a diagnosis. In order to determine the degree of extension of primary lesion, supplementary tests are used: cytoscopy, rectoscopy, urography, irigraphy, lung and bone radiography, lymphography, CT (MR) abdominal and pelvic, abdominal and pelvic ultrasound and laboratory analysis (Antic et al., 2012). Tumor markers in the past few years also take their place in the diagnosis of cervical cancer. Premalignant and malignant changes in the cervix may be associated with different risk factors. If it is longer and more frequent exposure to these factors, the possibility of development pathological changes on the cervix is bigger. For instance, in cervical cancer, p53 mutation is uncommon (Hietanen et al., 2000) but human papillomavirus (HPV) is present in more than 90% of the tumors (Schiffman et al., 2007). Harald zur Hausen identified the HPV in cervical cancer patients and in 2008, he received the Nobel Prize in Physiology in Medicine for his study of the etiology of cervix cancer and the role of HPV in the genesis of this disease. HPV is identified as the most significant individual risk factor and necessary condition for the development of cervical cancer (Schiffman et al., 2007). HPV types 16 and 18 are responsible for about 70% of all cases of cervical cancer in the world. HPV infection is the major risk factor of this disease, which, worldwide, is the second most common form of cancer in women. The HPV E6 protein complexes with cellular proteins E6-AP and p53 and facilitates p53 degradation via the ubiquitin dependent proteolytic system (Hietanen et al., 2000). On Figure 1 is shown HPV infection (transient infection and persistent infection) which leads to the invasive cervical cancer.

The relative risk of developing cervical cancer is about 65% higher in women with a positive finding of HPV as compared to those with the negative finding and also established that in women with chronic infection of HPV, there is a higher risk of progression of the normal Papa finding into the cervical intraepithelial neoplasia II or III (CIN II and CIN III) in a 2 year period (Al-Daraji and Smith, 2009), in relation to women with a negative finding of a human Papillomavirus (Schiffman et al., 2007; Al-Daraji and Smith, 2009; Singer and Khan, 2009). Recent researches show that a woman who is positive on infections of high-enzymatic HPV in two occasions after 6 and 12 months has the tendency to develop precancerous lesions of the cervix (Castellsagué et al., 2011). On the cervix, as well as on the other genital organs there is no sudden appearance of cancer lesion, but gradually several years there are certain changes in epithelial cells from which it will be possible to develop cancer later (Han et al., 2012). These changes can not be observed macroscopically, they can only be detected by microscopic examination of cells derived from a cervical or vaginal swab or by a clipping of suspicious tissue during a colposcopy examination (Hewitt et al., 2004). Changed cells of the cervical epithelium show less pronounced characteristics of malignant degeneration, they are unequal in size, irregular, nucleoprotoplasmic relationship is changed in favor of the nucleus, the nuclei are large, also irregular, hyperchromatic, non-homogeneous colored, cytoplasm is narrowed, poorly colored. The natural order of the cell layers from the basal membrane to the surface is disturbed and a higher percentage of cells in the mitosis is observed. These changes are less intense and do not involve all layers of the epithelium are designated as dysplasia-CIN (Saslow et al., 2002; Shastri et al., 2005). Cervical dysplasia can occur in three stages: lighter (CIN I), moderate (CIN II) and severe (CIN III-Ca *in situ*). Dysplasia of an easier degree (CIN I) means the finding of altered cells only in the lower third of the epithelium (Saslow et al., 2002).

In moderate degree of dysplasia (CIN II), disorder of stratification of the epithelium and the altered cells are observed from the basal layer to half of the epithelium, and in the third-degree dysplasia (CIN III-Ca *in situ*) altered epithelial cells and the stratification disorder will see to the surface of the epithelium (Sankaranarayanan et al., 2004; Shastri et al., 2005). It is thought that first and second degrees îf dysplasia may, after a certain time, spontaneously or under the influence of the therapy withdraw and to cure (which happens in most cases). At a small percentage, changes are maintained and certain number is malignantly altered (Sankaranarayanan et al., 2004). This can not be said for third-degree dysplasia, which is a diagnostic rather difficult to distinguish from preinvasive cancer (Saslow et al., 2002). As the third degree of dysplasia develops in time through the transition to preinvasive, and through this in invasive cervical carcinoma, when diagnosing this condition, surgical treatment is required (Al-Daraji and Smith, 2009).

Otherwise, cervical dysplasia, as well as preinvasive carcinoma of this organ, do not lead to visible macroscopic changes, although more often than usually they are detected in leukoplakia and erythroplakia of the cervix. Preinvasive cervical cancer is a stage of a malignancy in which malignant changes are localized only in the cervix epithelium, most often around the outer estuary of the uterus (Castellsagué et al., 2011).

In this case, the malignant cells have not yet broken through the basal membrane and thus not penetrated even in subepithelial tissue. Therefore, this stage of the disease is called intraepithelial, zero or carcinoma *in situ*. Since the initial malignant process is localized in the cervix epithelium itself and has not penetrated through the basal membrane into deeper tissues, there is no metastatic spreading of disease at this stage. The diagnosis of preclinical stage is not easy because the cervix is macroscopically with or without noticeable changes (erythroplakia, leukoplakia, disturbed vascular pattern). Therefore, the diagnosis of preinvasive carcinoma of the cervix is made during controlling routine or systematic gynecological examinations based on a positive cytological finding (III and IV group of cervical secretion by the Papanicolaou method) or on the basis of colposcopic examination (Janicek and Averette, 2001). It is best, if a conization is used for taking the sample, which is at the same time sufficient therapeutic measure in case of microscopic confirmation of the diagnosis of dysplasia or preinvasive carcinoma. Conization is a method of selecting in the treatment of young women with preinvasive cervical cancer, because it only removes part of the mucous membrane around the external estuary and along the cervical channel with a little bit of surrounding myometrium without damaging the internal organs and without disturbing their functions.

**FIGURE 2:**
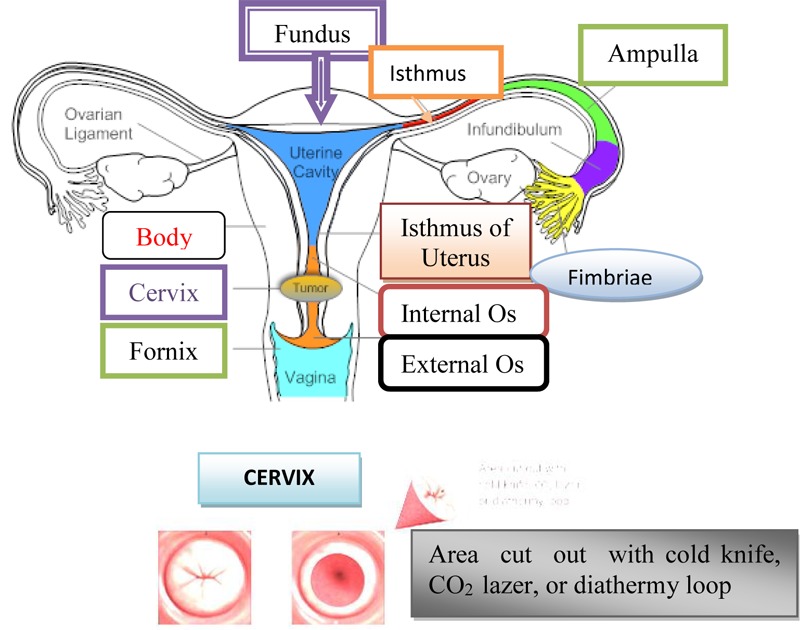
Cone biopsy (conization of the cervix).

Also, conization can be done in pregnant women, if preinvasive carcinoma is detected during pregnancy. In the case of carcinoma *in situ*, which diagnosed in older women, instead of conization should be performed a total hysterectomy. If CIN is not detected at this stage of preinvasive carcinoma and if carcinoma does not withdraw by therapy, what occurs at a high percentage in the early stage, the malignant process continues with development, which lasts for more than 10 years and the disease passes into an invasive stage (Mishra et al., 2011). On Figure 2 is shown Cone Biopsy (Conization of the Cervix).

The first period of this stage, while the invasion is less than 3 mm (so called microinvasion), also passes asymptomatic and macroscopic changes and other symptoms of the disease occur only in the second period of the first stage of the disease. Carcinoma of the cervix is the most common localization of the malignant process on the woman’s body immediately after the breast cancer. This malignant disease occurs in relatively young women, even in girls. It is interesting and very important that, thanks to increasingly comprehensive preventive measures, especially in developed countries with high living standards in recent decades, the number of cervical cancers in the invasive stage gradually and permanently disappears and slowly approaches the number of cases with endometrial carcinoma. This is enhanced by the improved personal health and hygiene culture of women and the environment in which they live, a better organized health service and greater care for the health of women, which involves primarily organizing periodic systematic researches of the female population at the time of the greatest vulnerability (Duval et al., 2009). In recent decades, at Papillomas and herpes viruses have been occurred an increasing importance due to the emergence of epithelial dysplasia, including neoplasia on the cervix (Al-Daraji and Smith, 2009). It is believed that under the influence of these viruses that usually penetrate in the epithelium at the site of the metaplasia of the plate-layered epithelium in the cylindrical formation, there are changes in the epithelial cells, which leads to the dysplasia of these cells. These changes can initially be reversible, but they can also develop into invasive carcinoma. The fight against viral infection or Papillomavirus infection is in fact the fight against the development of malignant neoplasia and other genital organs of the women (vulva, vagina). Cervical carcinoma usually begins around the outer mouth of cervix in place crossing plate-layered epithelium into a single-layer cylindrical endocervix epithelium (Hoffman et al., 2003).

Malignant changes first appear on the cells of the platelet epithelium around the outer mouth of the uterus and remain relatively in the epithelium itself, without penetrating immediately into the stroma, i.e., without causing invasive cancer.

This is the so-called preinvasive or intraepithelial stage of cervical cancer. At that time, changes in the epithelium can not yet be observed macroscopically. The disease at this stage remains for several months, even for several years. If the initial malignant lesion is not detected and immediately not removed at that stage, there will be further changes.

Spreading along the surface will lead to the erosion of the cervix, which can be seen by the naked eye and the penetration into depth will lead to invasion into the stroma and it may be expected metastases in the regional lymph glands and distant organs and infiltration of adjacent tissues and organs (Li et al., 2016; Xiaotian et al., 2017). The already established macroscopic erosion usually spreads around the external estuary and it can develop in three macroscopic types: vegetative, ulcerous and infiltrative (Xiaotian et al., 2017). The most common is a vegetative form when on the cervix are created friable growths that gives the appearance of cauliflower. At the macroscopic form of cervical cancer, there is a slow tendency toward the spread of the malignant process in depth and infiltration of the parametrium and vagina (Li et al., 2016). Significant macroscopic changes can develop on the vaginal portion of the cervix and the process is still in operative stage. The surface of the cervical cancer is elevated, uneven and dilapidated. When examining or touching it, small pieces of tissues drop off and from the damaged blood vessels occurs an easy, so called contact bleeding, which is also one of the first symptoms of this disease. The infiltrative form of cervical cancer, as its name suggests is characterized by relatively early penetration or infiltration of the malignant process into the deeper tissues, through the basal membrane to the cervix wall, into the parametrium and into the upper part of the vagina wall. The same applies to the ulcerous form that is besides rapid infiltration, followed by the formation of ulceration on the cervix, which infiltrates the surrounding tissues, destroys them, including in the first place the cervix and produces ulceration.

Both in infiltrative and in the case of ulcerative type, the surface of the resulting ulceration is friable, therefore the malignant change bleed to the touch. In these two forms and especially in the infiltrative form, the malignant process rapidly penetrates to deep tissues (Li et al., 2016). The vaginal portion of the cervix for the long time macroscopically remains unchanged, as the malignant process extends from the outer mouth of cervix, along the tissue of the endocervix and it is hidden from the gynecologist’s view in a regular gynecological examination.

In this case occurs to the malignant infiltration of the tissue from the endocervices to the deeper layers of the cervix. Cervix can be significantly changed, because it is invaded by malignant tissue (Li et al., 2016).

**FIGURE 3:**
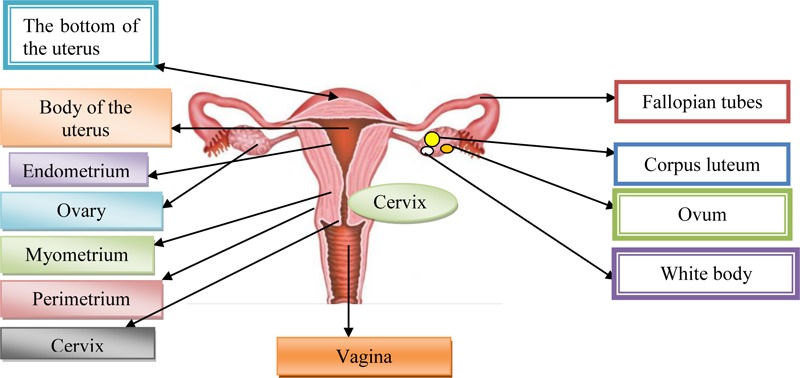
Showing a healthy cervix and cervix with carcinoma.

Therefore, in a digital examination, vaginal portion of the cervix looks sturdy, more or less enlarged and it is in advanced process usually driven harder. In addition, quite often the cervix gets a barrel shape. In any case at the endocervical carcinoma it is more difficult to place a timely diagnosis. That’s why this form of cancer is usually diagnosed later, so it’s also malignant in terms of flow and with worse prognosis (Comber and Gavin, 2004). By microscopic examination, cervical cancer is classified as carcinoma of the platelets and very rarely as the carcinoma of the cylindrical epithelium. The carcinoma of platelets epithelium can be a planocellular, basocellular and intermediate type. Less mature microscopic forms of cancer develop faster, they give metastases earlier and they have a worse prediction (Li et al., 2016). Rarely, if it is not endocervical localization in question, where the cylindrical glandular epithelium is normally found, occurrence of this histological form of the cancer on the outer surface of the cervix is preceded by a metaplasia of the plate-layered in the cylindrical epithelium (Li et al., 2016). In any case, that should be known that cervical cancer is of a cylindrical type cell resistant on radiation treatment and that whenever possible it is desirable to initiate operative therapy first (Janicek and Averette, 2001). Regardless of the choice of treatment, the prognosis is less favorable than at the carcinoma of plate-layered epithelium. On Figure 3 is shown a healthy cervix and cervix with carcinoma.

Cervix uterus, Cx is the lower fibrous-muscle part of the uterus, cylindrical or conical, with a length of 3–4 cm, and an average diameter of 2.5 cm (American Joint Committee on Cancer [AJCC], 2017). The cardinal and sacrouterine ligaments extend between the lateral and posterior part of the cervix and the pelvic wall. The lower half of the cervix is called vaginalis uteri, PVU is located in the vagina, while its upper half is located above the vagina/supravaginal cervix. We distinguish on PVU two lips: front and rear, and between them there is an external uterine mouth, which represents the place where the cervical channel opens into the vagina (American Joint Committee on Cancer [AJCC], 2017). The dimension and shape of the cervix depend on the age, parity and menstrual or hormonal status of women. In women, who have not given birth, the cervix is small and the outer is seen as small, central, circular opening. Supravaginal part of the cervix continues in the muscular part of the body uterus, in conjunction with the channel isthmus of the uterus, and at its upper end exist internal mouth.

A part of the cervix that is externally from the external uterus is called ectocervix and that is the part of the cervix that is immediately noticed during the examination under the speculum. Part of the cervix proximal from the outer mouth of the uterine called endocervix. The endocervical channel extends from the outer to the inner uterine mouth and passes through the entire length of the endocervix and connects the uterine cavity and vagina (American Joint Committee on Cancer [AJCC], 2017).

## Diagnosis of Invasive Stage of Cervical Cancer

**FIGURE 4:**
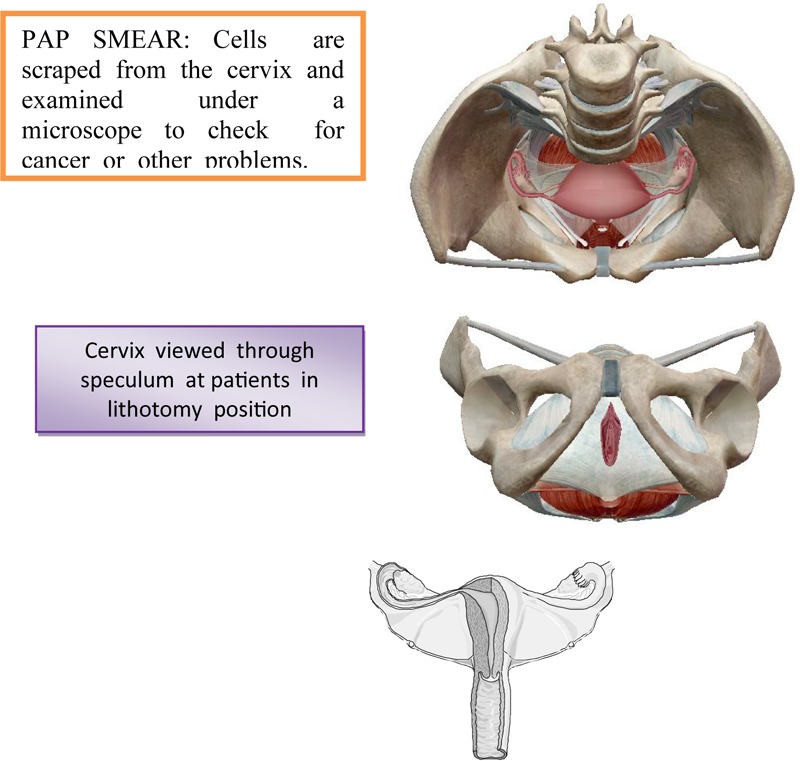
Taking a swab from the cervix through the Papanicolaou test.

The diagnosis of the invasive stage is set by clinical examination, supplemental examinations and laboratory analyzes (Bayo et al., 2002). Each examination could be initiated by reviewing the cervix by means of vaginal ecarter or speculum. There is less or greater erosion on the cervix around the external estuary of the uterus (Hewitt et al., 2004). From the last vaginal vestibule and from the outer estuary of the uterus, the secretion is taken by the swab and the smear, which is colored by the Papanicolaou method is examined using a microscope (Arnheim Dahlström et al., 2011). On the Figure 4 is shown a taking swab from the cervix through the Papanicolaou test.

The material for cytological examinations can also be taken from the cervical channel and from the surface of the cervix and from its outer mouth using a special spatula or aspiration. Using Papanicolaou-colored method, it can notice the finding of large, unequal, irregularly shaped cells, whose irregular and unequal hyperchromatic cores point to malignancy.

**FIGURE 5:**
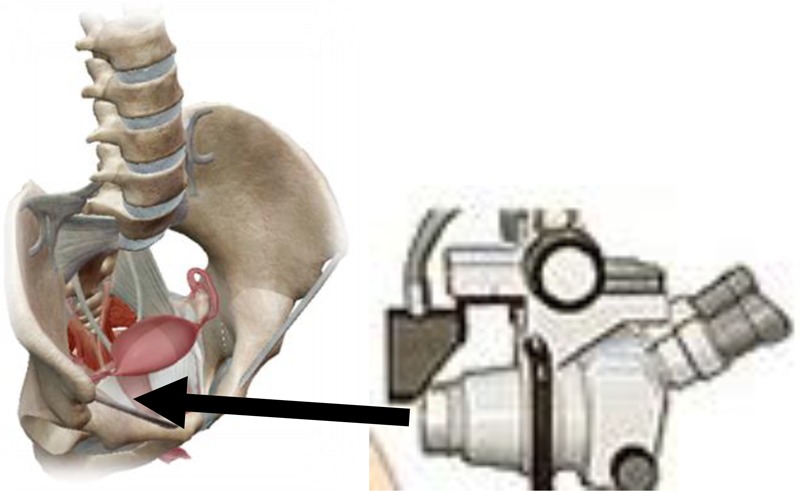
Colposcopy as a primary screening test for cervical cancer.

In addition to examining of the vaginal smear in the diagnosis of invasive cervical cancer is used colposcopy (Bayo et al., 2002). The definitive diagnosis of cervical cancer is made by a histological examination of cervix by cutting out of obtained materials. The clipping from the cervix should be taken from a place that appears under the ecarter as the most suspected place, where the initial malignant lesion may exist (Janicek and Averette, 2001). Sometimes, therefore, instead of one, the material for microscopic examination is taken from two or even three places (Hricak et al., 2005). Great help in determining the site from which the clipping should be taken can provide coating of the cervical surface with an iodine tincture or colposcopy. Such a biopsy, or taking a snippet from a suspected site, defined by Schiller’s iodine test or colposcopy is called a target biopsy. In any case, the great importance of the early diagnosis of cervical cancer has a mandatory examination of a woman under the ecarters by using a speculum before a bimanual examination (Hricak et al., 2005). On Figure 5 is shown a colposcopy as a primary screening test for cervical cancer.

There are no early symptoms of cervical cancer, which would alert the patient to the existence of the disease at a pre-invasive stage (Shastri et al., 2005; Epstein et al., 2013). When the symptoms occur, the disease has already become more severe, it is better to say that it has been a long time in the invasive stage. Therefore, instead the appointment “early” use appointment “first symptoms” of the disease (Epstein et al., 2013). There are few symptoms and they are not always alarming, so patients often do not attach importance to them, which they deserve in a diagnostic view. Early or better to say the first symptoms of cervical cancer include: contact bleeding and persistent bleeding secretion, resistant on the therapy (Hricak et al., 2005). There are the symptoms, only when the necrosis of the superficial epithelium of the malignant lesion occurs, in fact, when an invasion of the malignant process has already occurs in the subcellular stroma and when there is the possibility and likelihood that the lymphatic pathways and adjacent lymph nodes are already covered by malignant infiltration (Xiaotian et al., 2017). Bleeding is usually scarce and occurs after sexual intercourse or gynecological examination. Although contact bleeding can also occur in other diseases and changes in female genital organs, especially on the cervix, whenever it is observed it should be taken seriously and gynecological examination and other supplementary measures should be asked for its true cause and therefore the exact diagnosis (Hricak et al., 2005).

In this review, it can detect susceptible erosion, whose character needs to be examined in further research. If, it is not done, the disease will continue to progress, there will be necrotic changes on the surface of the malignant lesion, resulting in the secretion of the bleeding component. For this reason, a bleeding secretion with the features of extract washed out of meat, indicates on the existence of cervical cancer, usually in an already advanced stage (Shastri et al., 2005). The third sign-pain occurs quite late, when the disease has significantly gone and when the malignant infiltrate penetrates deeply into the parametrium and pelvic bones. For the success in the treatment, it is crucial to set the diagnosis before the appearance of pain in the small pelvis and the spine.

## Clinical Stages of Cervical Cancer

The FIGO (International Federation of Gynecology and Obstetrics) staging system is used most often for cancers of the female reproductive organs, including cervical cancer. For cervical cancer, the clinical stage is used and is based on the results of the doctor’s physical exam, biopsies, imaging tests, and a few other tests that are done in some cases, such as cystoscopy and proctoscopy. It is not based on what is found during surgery. If surgery is done, a pathologic stage can be determined from the findings at surgery, but it does not change your clinical stage. The treatment plan is based on the clinical stage (Benedet et al., 2000).

Stage I is carcinoma strictly confined to the cervix; extension to the uterine corpus should be disregarded. The diagnosis of both Stages IA1 and IA2 should be based on microscopic examination of removed tissue, preferably a cone, which must include the entire lesion (Benedet et al., 2000).

IA: Invasive carcinoma that can be diagnosed exclusively by microscopic. All macroscopically visible lesions, even very small, are noticed in stage I. It’s an invasion limited to a depth of maximum 5 mm, and a horizontal spread of maximum 7 mm. The depth of the invasion must not exceed 5 mm, measured from the base of the epithelium of the original tissue, surface or glandular. The invasion of vascular spaces, blood and lymphatic, does not change the stage of the disease (Di Stefano et al., 2005).

IA1: Limited to the cervix, diagnosed exclusively by microscopic, the depth of invasion of the strome ≤ 3 mm, horizontal expansion ≤ 7 mm (Hacker, 1988).

IA2: Restricted to the cervix, diagnosed exclusively by microscopic, the depth of invasion of the strome > 3 mm, or ≤ 5 mm, horizontal spread ≤ 7 mmIB Clinically visible lesions limited to the cervix, or microscopically detected tumors larger from the stage IA.

IB1: Clinically visible lesion or microscopic lesion > IA2, but < 4 cm in the largest diameter.

IB2: Clinically visible lesion, > 4 cm in the largest diameter (Hacker, 1988; Di Stefano et al., 2005).

Classification of cervical cancer as well as all changes to any of genital organs, according to the progression of the process are grouped into four stages (Benedet et al., 2000). In Table 1 is shown division of tumor according to TNM category and FIGO stadium.

**Table 1:** Division of tumor according to TNM category and FIGO stadium.

Primary tumor	TNM category	FIGO stadium
The primary tumor cannot be estimated	T_x_	
Without primary tumor	T_0_	0
Minimal microscopic stromal invasion	T_1_a_1_	Ia_1_
Stromal invasion up to 5 mm in depth from the epithelial base and up to 7 mm in horizontal spread	T_1_a_2_	Ia_2_
Tumor greater than T_1_a_2_	T_1_b	Ib
Invasion of the upper third of the vagina	T_2_a	IIa
Invasion of internal 2/3 parametrium	T_2b_	IIb
Invasion of the lower third of the vagina without spread to the wall of the pelvis	T_3_a	IIIa
Invasion of the parametrium to the wall of the pelvis and/or causes a hydronephrosis or a irregular kidney function	T_3_b	IIIb
Invasion of mucous membranes of the bladder or rectum and/or spreads beyond the pelvis	T_4_	IVa
Distant metastases	M_1_	IVb

Stage II is carcinoma that extends beyond the cervix, but does not extend into the pelvic wall. The carcinoma involves the vagina, but not as far as the lower third.

Stage IIA: No obvious parametrial involvement. Involvement of up to the upper two-thirds of the vagina.

IIA-1: Clinically visible lesion < 4.0 cm in greatest dimension.

IIA-2: Clinically visible lesion > 4.0 cm in greatest dimension (Kashima et al., 2010).

Stage IIB: Obvious parametrial involvement, but not into the pelvic side wall. Stage III is carcinoma that has extended into the pelvic sidewall. On rectal examination, there is no cancer-free space between the tumor and the pelvic sidewall. The tumor involves the lower third of the vagina. All cases with hydronephrosis or a non-functioning kidney are Stage III cancers (Kashima et al., 2010).

Stage IIIA: No extension into the pelvic sidewall but involvement of the lower third of the vagina.

Stage IIIB: Extension into the pelvic sidewall or hydronephrosis or non-functioning kidney.

Stage IV is carcinoma that has extended beyond the true pelvis or has clinically involved the mucosa of the bladder and/or rectum.

Stage IVA: Spread of the tumor into adjacent pelvic organs.

Stage IVB: Spread to distant organs (Benedet et al., 2000).

The American Joint Committee on Cancer (AJCC)TNM staging system is another staging T describes how far the main (primary) tumor has grown into the cervix and whether it has grown into nearby tissues.

N indicates any cancer spread to lymph nodes near the cervix. Lymph nodes are bean-sized collections of immune system cells, to which cancers often spread first system based on 3 key pieces of information:

M indicates if the cancer has spread (metastasized) to distant sites, such as other organs or lymph nodes that are not near the cervix.

The US Committee for Cancer proposed the TNM classification, based on the extension of the primary tumor (T), the existence of metastases in lymph nodes (N) and the existence of distant metastases (M). TNM classification also involves determining the involvement of regional lymph nodes (N), which include: paracervical, parametric, obturatorial, external, internal and common ilical and sacral lymph nodes (Benedet et al., 2000). Metastases in paraaortal lymph nodes are considered distant metastases. FIGO and TNM classification are basically the same, and the disease stages are comparable in mentioned classifications. Special attention should be paid to the fact that according to the AJCC classification is present metastasis in regional lymph nodes, which according to TNM classification corresponds to N1, translates stage of disease according to FIGO classification in stage IIIB (Benedet et al., 2000).

Cervical cancer can be spread in the following ways:

• Direct invasion of the cervical stroma, the body of the uterus, the vagina and the parametrium
• Lymphatic permeation and metastasis.
• Hematogen dissemination

### Direct Infiltration

Invasive carcinoma, squamous or glandular, arises from intraepithelial neoplasia. Malignant cells break through the basal membrane and progressively infiltrate the stroma that lies beneath the basalmembranes. The lateral infiltrating cardinal and sacrouterine ligament can progressively expand, proximal, infiltrating endometrium, distal, infiltrating the vagina, anteriorly infiltrating urinary bladder and posterior, infiltrating Douglas spag and rectum (Benedet et al., 2000).

### Lymphogenic Spread

Cervix can spread to all groups of pelvic lymph nodes, but obturator nodules most often affected. Parametrial nodes are more rarely positive than nodes in iliacal obturator cave. Parametrial nodes are more rarely positive than nodes in iliacal obturator cave. Spreading into common iliacal nodes can be direct, although very rare through the last cervical lymphatic pathway and it is often affected by the spread of metastatic changes nodes of the iliac and obturatoric regions. Paraaortal nodes can be drained over the ductus thoracicus in supraclavicular nodules, although this type of metastasis is rare (Altgassen et al., 2008).

### Hematogenic Spread

Hematogenic spread. Hematogenic spread is possible in any part of the body, the most commonly spread of cervical cancer is in the lungs, liver, and bones. Rarely, the intestine, adrenal glands, spleen and brain may be affected (Benedet et al., 2000).

In Table 2 is shown presence and absence of metastases in regional lymph nodes and distant metastases.

**Table 2:** Presence and absence of metastases in regional lymph nodes and distant metastases.

N	Regional lymph nodes
Nx	Regional lymph nodes cannot be evaluated
No	Without metastases in regional lymph nodes
N_1_	Metastases in regional lymph nodes
Mx	The presence of distant metastases cannot be estimated
M_0_	Without distant metastasis
M_1_	Distant metastases

Lymph nodes play an important role in cancer staging, which determines the extent of cancer in the body. One of the most commonly used systems for staging cancer is the TNM system, which is based on the extent of the tumor (T), the extent of spread to the lymph nodes (N), and the presence of metastasis (M) (Altgassen et al., 2008). Treatment for cancer in the lymph nodes depends on a variety of factors, including tumor size and location, and whether or not the cancer has metastasized (spread) to other areas of the body (Altgassen et al., 2008). The spread of cervical cancer to the uterine body has no importance for TNM, nor for FIGO staging (Benedet et al., 2000).

The division into stages is mainly based on a clinical finding, established by inspection, palpation, colposcopy, endocervical curettage, X-ray examination of the lungs and skeletons and urography. Also, conization and amputation of the cervix can be used in the detection of cervical cancer and contribute to its classification (Han et al., 2012). Coincidentally detect hot spots of cervical cancer in the preparation obtained in hysterectomy surgery, may have significance in the assessment of disease progression. Classification of cervical cancer refers only to the primary malignant neoplasm of this organ, regardless of the histological type of tumor. Cervical cancer can be divided into two major categories: preclinical or preinvasive stage and clinical or invasive carcinoma. The preclinical stage is characterized by the onset and initial development in the disease in the epithelium of the cervix without penetration through the basal membrane and the passage of the malignant process in stroma (Altgassen et al., 2008).

Therefore, at this stage, there is no risk of transmitting malignant cells to regional lymph nodes and distant organs. For the preclinical stage of disease, the name CARCINOMA *IN SITU* is known (Han et al., 2012). At this stage, it occurs a very early, macroscopic invisible lesion in the mucous membrane of the cervix. The evolution of preinvasive into the invasive stage is very gradual and it lasts for 10 years. It should not be emphasized that the preinvasive form of the cancer is very grateful for the treatment and in this stage 100% successful results are achieved (Han et al., 2012).

The invasive form of cervical cancer is clinically agreed upon by the International Federation of Gynecologists and Obstetricians in four stages. The International Association of Gynecologists and Obstetricians (FIGO) suggests the use of magnetic resonance imaging (MRI) during determining the stage of cervical cancer (Benedet et al., 2000). When a decision on a definitive therapeutic modality is made, MRI is used as a substitute for invasive techniques such as cystoscopy and endoscopy in the evaluation of the extension of the tumor process to the urinary bladder and rectum. When determining the stage of cervical cancer, MRI has the role to determine the size and localization of the tumor lesion, the presence of an invasive process in the surrounding structures, and the existence of metastatic focuses including increased lymphoid glands in small pelvis and abdomen (Benedet et al., 2000). As a completely non-invasive diagnostic method, MRI has multiple role in the diagnosis of cervical cancer, enabling determination of the degree of disease spread and prognostic factors crucial for therapy planning, monitoring of the effects of therapy and diagnostics of recurrence (Altgassen et al., 2008).

Stage I of cervical cancer implies the presence of cancer that is limited to the cervix. Stage II includes tumors, with the penetration of the vagina and parametria (Altgassen et al., 2008). The advanced stage III denotes the penetration of the tumor to the pelvic walls, the presence of kidney hydronephrosis or invasion of the lower third of the vagina while stage IV involves the presence of distant metastases out of the pelvis or infiltration of the mucous membrane of the urinary bladder and rectum (Altgassen et al., 2008).

### First Stage of Cervical Cancer

**FIGURE 6:**
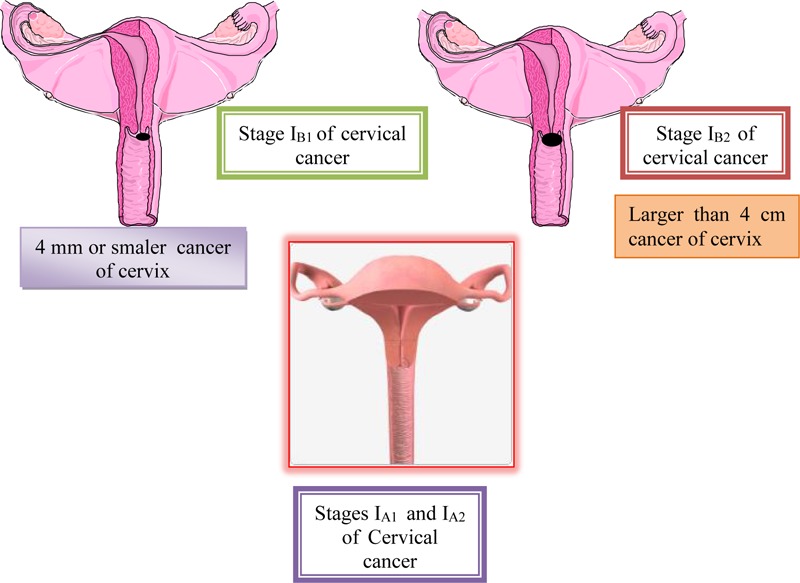
I_A1_ I_A2_, I_B1_, and I_B2_ stages of cervical cancer.

The first stage of the disease involves cases in which the malignant change is strictly limited to the cervix. This stage is divided into two subgroups. At Ia stage, cancer can not be diagnosed with a regular examination, but only based on laboratory analysis. First stage includes early-invasive carcinoma and occult carcinoma. It is divided into two subgroups Ia1 and Ia2 (Zhilan et al., 2016). In the first subgroup, the invasion of stroma into depth reaches up to 5 mm and horizontally up to 7 mm. As in the Ia stage, although theoretically possible, metastases in regional lymph nodes are exceptionally rare, certain clinicians (Mestvert from Germany) have introduced in clinical practice the notion of microinvasive carcinoma, which in cervical cancer causes malignant lesion, which can only be detected by microscopic examination and whose depth of invasion process and the therapy should be limited to conization or total hysterectomy without radical surgery. After the intervention, the patient must be kept under regular control for a long time. Stage Ib includes all other cases of the first stage of cervical carcinoma, or cases when the malignant change in the form of erosion is clearly visible on the cervix, but when there is no macroscopic infiltration of the vagina or parametrium with malignant neoplasmic tissue (Zhilan et al., 2016). On Figure 6 is shown I_A1_, I_A2_, I_B1_, and I_B2_ stages of cervical cancer (Morice et al., 2000).

### Second Stage of Cervical Cancer

**FIGURE 7:**
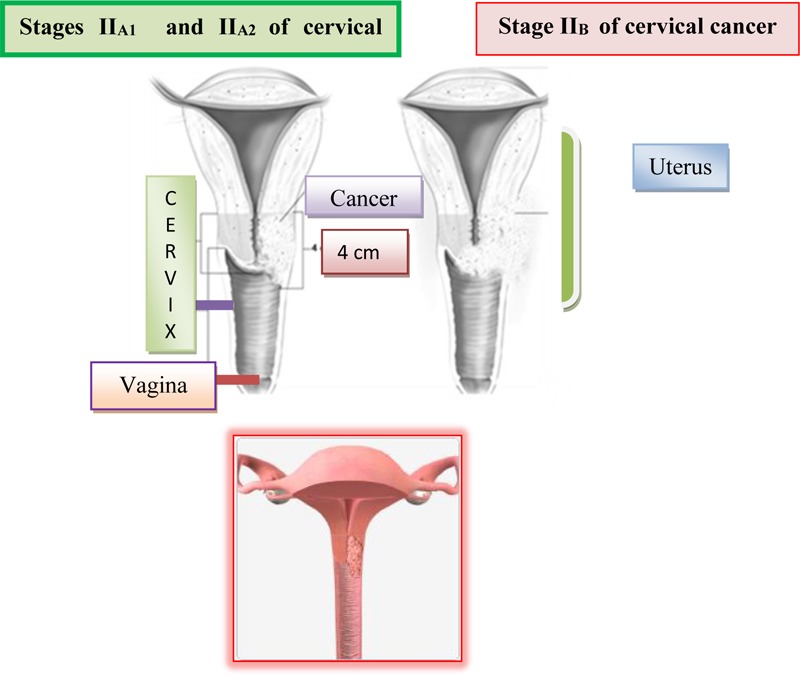
Stages II_A1_, II_A2_, and II_B_ of cervical cancer.

The malignant infiltrate is clearly spreading beyond the cervix, i.e., it permeates the wall of the cervix and passes to the nearest environment, the upper third of vagina and one part of parametrium. The uterus is still partially free and limited in movement, because the malignant infiltrate has not yet reached to the pelvic bone through the parametrium. The malignant process involves these most part of cervix and the likelihood of metastatic occurrence in the surrounding lymph nodes is significantly higher. At stage IIa, the malignant process has crossed on the upper third of vagina, whereby the lower two thirds are free and parametrium is not yet infiltrated. In stage IIb, the malignant infiltrate, in addition to the vagina, also affected the internal two thirds of parametrium, which are shortened and whereby the mobility of the uterus is limited (Zhilan et al., 2016). On Figure 7 is shown stages II_A1_, II_A2_ and II_B_ of cervical cancer.

### Third Stage of Cervical Cancer

**FIGURE 8:**
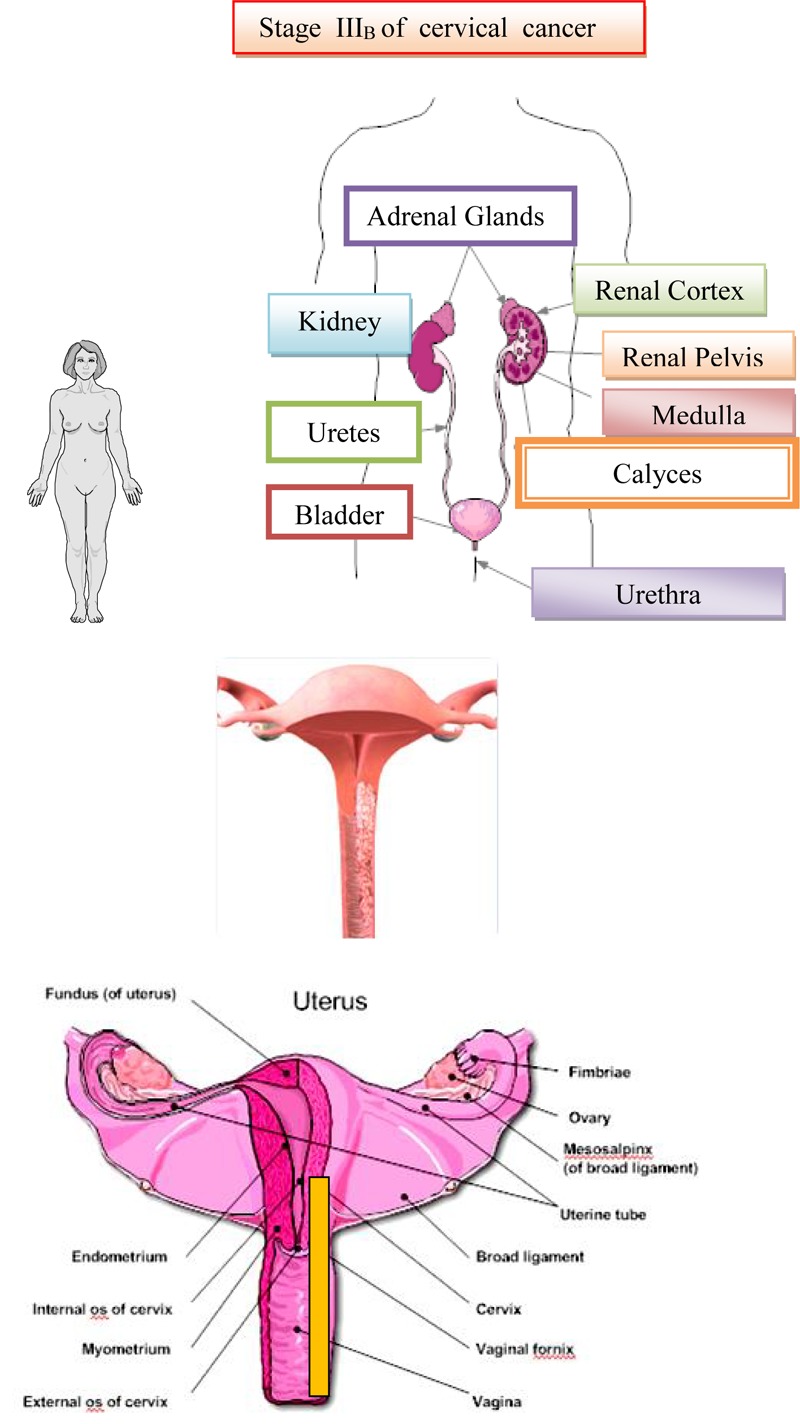
Stages IIIA and III_B_ of cervical cancer.

In the IIIa stage, the invasion spreads to the lower third of vagina and the infiltrate does not reach to the wall of the small pelvic and in the IIIb stage it passes to the pelvic wall and possibly leads to hydronephrosis. The cervix is almost completely affected by a malignant infiltrate, which across parametrium infiltrates the entire width, reaches to the pelvic walls and engages the lower half of vagina. In the rectal examination, there is no free space between the tumor and the small pelvic wall. Because of this, the uterus is surrounded by surrounding infiltrate and it is only partially mobile or not mobile at all. The patients have severe pain in the lower abdomen and loins (Li et al., 2016). The surrounding and distant lymph nodes are affected with metastasis of malignant tissue. On Figure 8 is shown Stages IIIA and III_B_ of cervical cancer.

### Fourth Stage of Cervical Cancer

**FIGURE 9:**
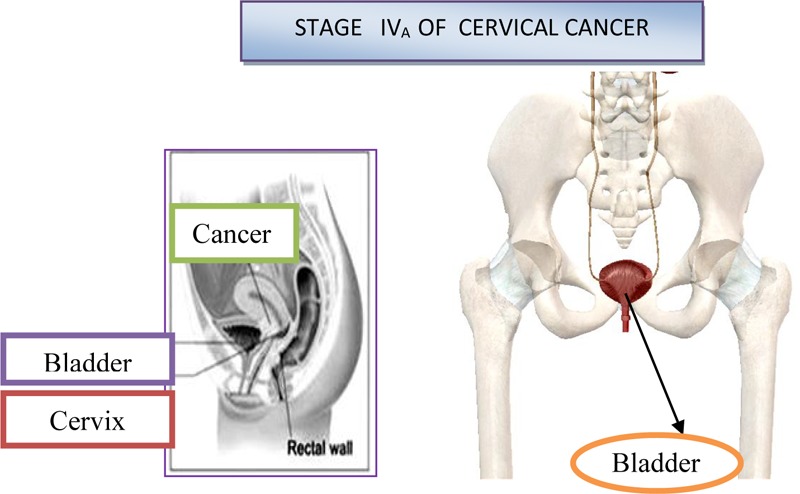
Stage IV_A_ of cervical cancer.

In the IVa stage, the malignant process spread out of the small pelvic, or it affected the mucous membrane of the urinary bladder or rectum. Bullous edema, by itself, if don’t exist other signs of the process, which cross on the bladder wall, doesn’t give the right that the particular case be classified in the fourth stage. On Figure 9 is shown stage IV_A_ of cervical cancer (Li et al., 2016). At this stage, the malignant infiltrate permeates the walls of adjacent hollow organs, so it can necrotize and create fistulas and unnatural communications between the urinary bladder, vagina and the rectum. In addition, in the IVb stage, the malignant process extends beyond and above the small pelvic to other organs of the abdominal cavity, giving distant metastasis. The patient is in miserable condition, cachectic with severe pain in the lower abdomen and loins. About the specific length of the disease debates by the consilium, in order to determine the stage of the disease and to make a decision about the method of the treatment. The consilium consists of experienced doctors-specialists: gynecologist, radiologist and oncologist.

**FIGURE 10:**
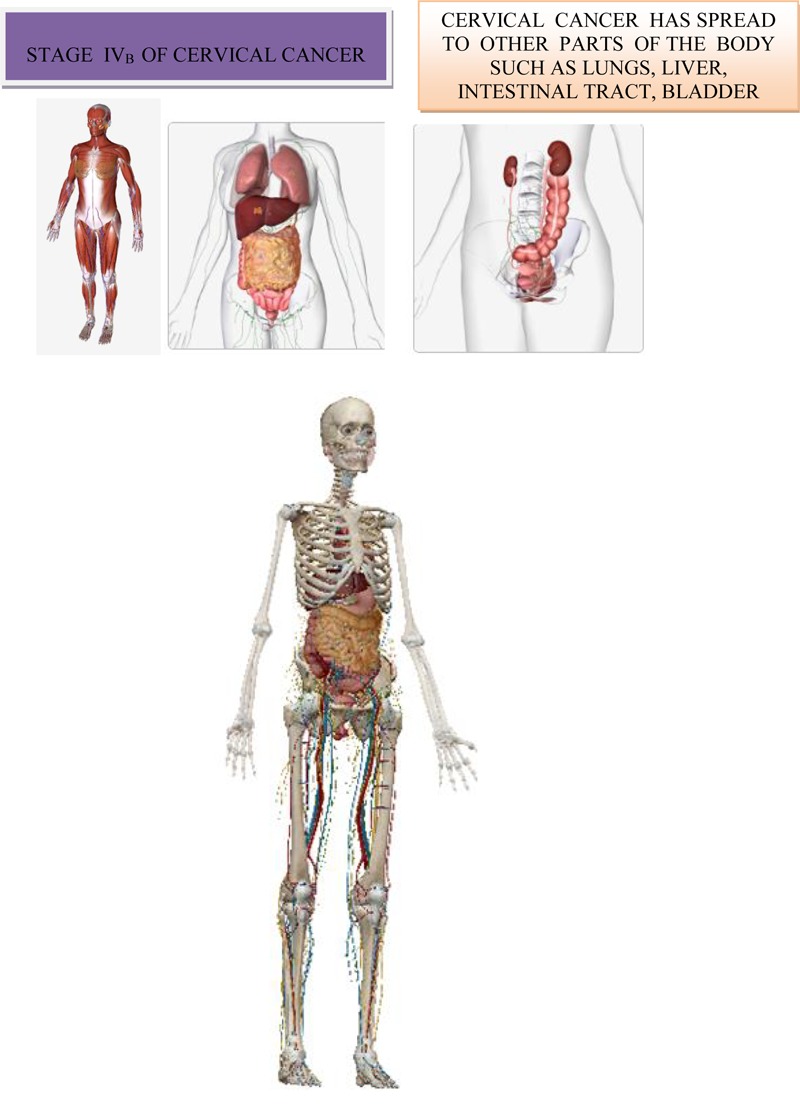
Stage IV_B_ of cervical cancer.

On Figure 10 is shown stage IV_B_ of cervical cancer (Li et al., 2016).

### Staging of Cervical Cancer

Cervical cancer staging is the assessment of cervical cancer to decide how far the disease has progressed. Cancer staging generally runs from stage 0, which is pre-cancerous or non-invasive, to stage IV, in which the cancer has spread throughout a significant part of the body.

Cervical cancer is staged by the International Federation of Gynecology and Obstetrics (FIGO) staging system, which is based on clinical examination, rather than surgical findings. It allows only the following diagnostic tests to be used in determining the stage: palpation (feeling with the fingers), inspection, colposcopy, endocervical curettage, hysteroscopy, cystoscopy, proctoscopy, intravenous urography, and X-ray examination of the lungs and skeleton, and cervical conization (Al-Kalbani et al., 2012).

• Stage 0: cervical intraepithelial neoplasia (HSIL or CIN III)
• Stage I: confined to cervix
• Stage Ia: invasive carcinoma only diagnosed by microscopy.
• Ia1: stromal invasion < 3 mm in depth and < 7 mm in extension (*microinvasive*).
• Ia2: stromal invasion > 3 mm depth and not > 5 mm and extension < 7 mm.
• Stage Ib: clinically visible lesions limited to the cervix or pre-clinical cancers > stage 1a.
• Ib1: clinically visible tumor < 4 cm in greatest dimension.
• Ib2: clinically visible tumor > 4 cm in greatest dimension (Kenter et al., 1989; Morice et al., 2000;Zivanovic et al., 2008).
• Stage II: beyond cervix though not to the pelvic sidewall or lower third of the vagina.
• Stage IIa: involves upper 2/3rd of vagina without parametrial invasion.
• Stage IIa1: clinically visible tumor < 4 cm in greatest dimension.
• Stage IIa2: clinically visible tumor > 4 cm in greatest dimension (Kenter et al., 1989; Morice et al., 2000).
• Stage IIb: with parametrial invasion (Kenter et al., 1989).
• Stage III.
• Stage IIIa: tumor involves the lower third of the vagina with no extension to pelvic sidewall
• Stage IIIb: extension to pelvic side wall or causing obstructive uropathy, MR imaging findings that are suggestive of pelvic sidewall involvement include tumor within 3 mm of or abutment of the internal obturator, levator ani, and pyriform muscles and the iliac vessel (Al-Kalbani et al., 2012).
• Stage IV: extension beyond true pelvis or biopsy proven to involve the mucosa of the bladder or the rectum
• Stage IVa: extension beyond true pelvis or rectal/bladder invasion
• Stage IVb: distant organ spread (Al-Kalbani et al., 2012).

#### Primary Tumor (T)

• Tx: Primary tumor cannot be assessed
• T0: No evidence of primary tumor
• Tis: Carcinoma *in situ*
• T1: Cervical carcinoma confined to the uterus
• T1a: Invasive carcinoma diagnosed only by microscopy
• T1b: Clinically visible lesion confined to the cervix
• T2: Cervical carcinoma invades beyond uterus but not to pelvic wall or to lower third of vagina
• T2A: Tumor without parametrial invasion
• T2B: Tumor with parametrial invasion
• T3: Tumor extends to pelvic wall and/or involves lower third of vagina, and/or causes hydronephrosis
• T3a: Tumor involves lower third of vagina, no extension to pelvic wall
• T3b: Tumor extends to pelvic wall and/or causes hydronephrosis
• T4: Tumor invades bladder or rectum, and/or extends beyond true pelvis

#### Regional Lymph Nodes (N)

• Nx: Regional lymph nodes cannot be assessed.
• No: No regional lymph nodes metastatsis.
• N1: Regional lymph node metastases (Di Stefano et al., 2005).

#### Distant Metastasis (M)

• M0: No distant mets.
• M1: Distant mets (including peritoneal spread, involvement of supraclavicular, mediastinal or para-aortic lymph nodes, lung, liver or bone).

In Table 3 is shown the staging of cervical cancer (Di Stefano et al., 2005).

**Table 3:** Staging of cervical cancer.

Stage	0	I	II	III	IV
Extent of tumor	Carcinoma *in situ*	Confined to cervix	Disease beyond cervix but not to pelvic wall or lower 1/3 vagina	Disease to pelvic wall or lower 1/3 vagina	Invades bladder, rectum, or metastasis
5-year survival	100%	85%	65%	35%	7%
Stage at presentation		47%	28%	21%	4%

**FIGURE 11:**
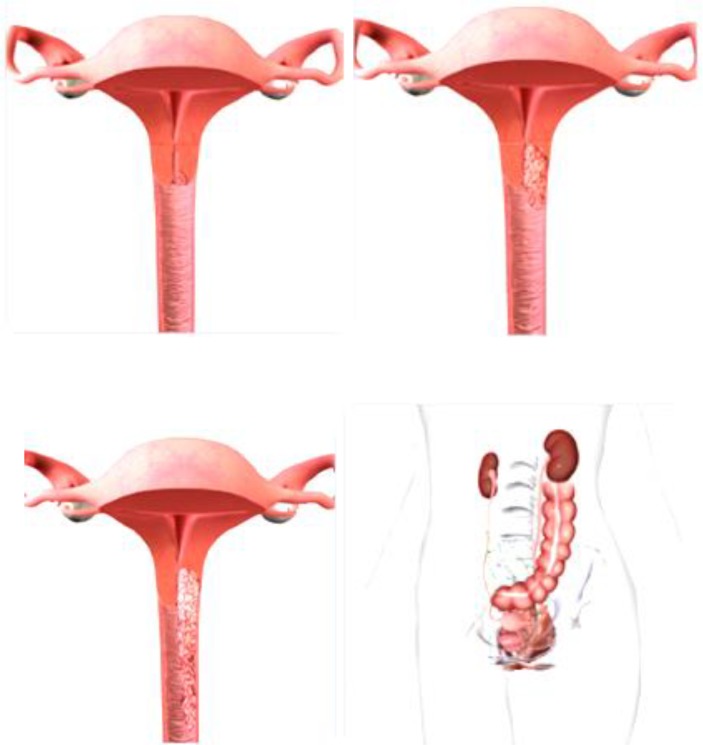
Four stages of cervical carcinoma.

On Figure 11 is shown a four stages of cervical carcinoma.

## Cervical Cancer and Pregnancy

Cervical cancer occurs most commonly in middle-aged women, but also in women in full-maturity, when they are capable to remain pregnant. Therefore, it is not particularly rare, if it is not a too advanced malignant lesion, that women, which suffering from cervical cancer get pregnant (Basta et al., 2002). Symptoms of cervical cancer in pregnancy are the same as outside gravidity. However, the illness is usually more difficult and reveals later, as even bleeding and increased secretion are often attributed to pregnancy, not to malignant process. Therefore, it is necessary that every pregnant woman, as well as other gynecologic patients be examined using a speculum or retractor and other diagnostic laboratory methods are also used. It should be kept in mind that the cytological and even histological findings in pregnancy can be somewhat altered without malignancy, which can mislead a cytologist or histopathologist. From this reason, when referring materials to the laboratory, it should always be noted that it is a pregnant woman (Basta et al., 2002).

If it is suspected that there is a malignant process on the cervix in pregnant women, it is very important to set the exact diagnosis as early as possible, because it is noticed that cervical cancer is developed much more rapid in pregnant women than otherwise (Basta et al., 2002).

It has been observed in such cases that the pregnancy develops normally, until the time when the childbirth should be happening. If the primary hotspot is advanced, the dilatation of the cervical channel is difficult and sometimes even disable. However, the pregnancy is rarely brought to the end, except in undiagnosed cases (Basta et al., 2002).

If the malignant process is detected earlier in the course of pregnancy, then spontaneous childbirth is not awaited, but the patient undergoes either radical operative therapy, whereby the uterus being removed along with fetus or in the advanced stage of disease, the pregnancy is operatively completed and the patient refers to ionizing radiation (Basta et al., 2002). This is important to do, especially because pregnancy has a very adverse effect on the malignant process, that is, accelerates and exacerbates it. Pregnancy is maintained to the end at the women, who have cervical cancer, before the date of childbirth, when the fetus is capable for extrauterine life. In such cases, if occurs to spontaneously childbirth on the diseased cervix, usually big cleavages are created from which the patient abnormally bleeding. In most of these patients, complete dilatation of the cervical channel and the external estuary of the uterus is impossible, so the childbirth must be completed by cesarean section. Likewise, the childbirth is completed by cesarean section in pregnant women in whom the disease is detected in the last months of pregnancy, when the fetus is already capable for extrauterine life (Basta et al., 2002). If at pregnant woman is detected the preinvasive carcinoma of the cervix, then it is enough to do just conization and leaves gestation to the end. To ensure pregnancy from premature dilatation of the cervix or from spontaneous abortion during conization, it is necessary to consider the need to place the cerclage. After childbirth, such patient should be monitored for a long time (Basta et al., 2002).

## Treatment of Cervical Cancer

The choice of cervical cancer treatment depends on several factors-stage of disease, histopathological type of tumor, regional and distant metastases, degree of tumor differentiation (grade-G), primary lesion size, way of primary tumor growth, age and general condition of the patient. Today, three therapeutic modalities are used: surgery, radiotherapy, hormone chemotherapy (Janicek and Averette, 2001). Nevertheless, the first two modes dominate independently or in combination. Surgery as standalone and only therapeutic approach is used in preinvasive (Ca *in situ*) and microinvasive stage (stage Ia) of cervical cancer. Surgery and radiation are combined in Ib and IIa stages and only radiation is used in IIb, IIIa, IIIb and IVa. In stage IVb, (cervical cancer and distant metastases) are used chemotherapy and locoregional radiotherapy (Eaker et al., 2001).

**FIGURE 12:**
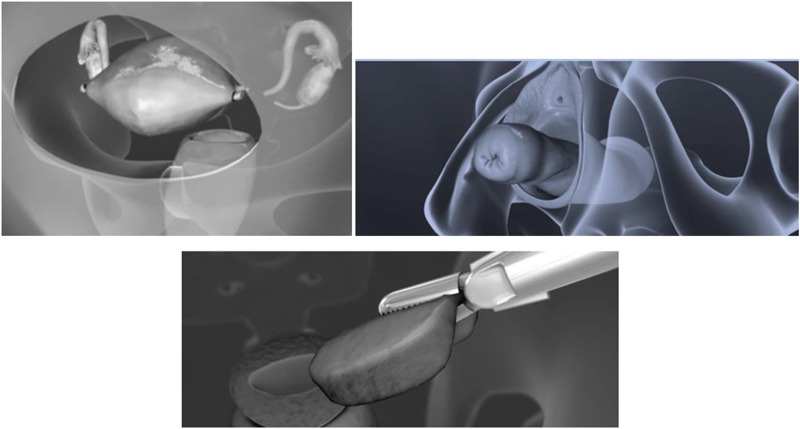
Abdominal, vaginal and laparoscopic hysterectomy.

**FIGURE 13:**
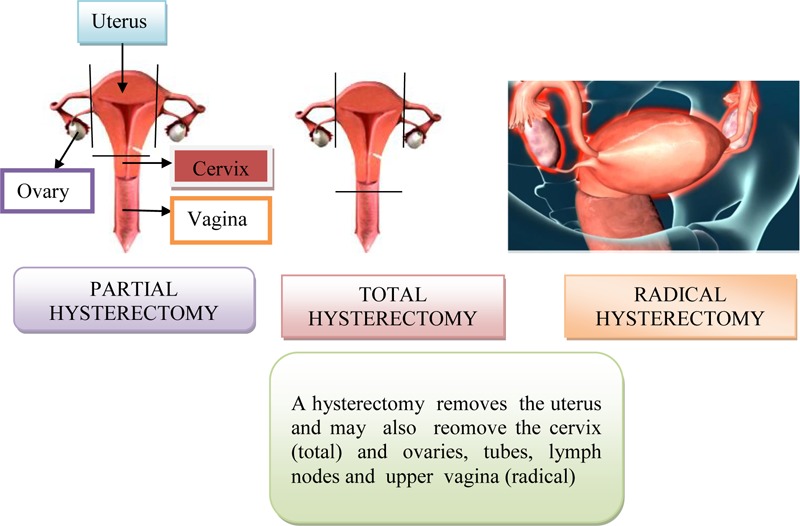
Radical hysterectomy for early stage of cervical cancer.

Surgical treatment initially consisted from transvaginal hysterectomy and then from transabdominal removal of the uterus (via laparactomy). On Figure 12 is shown abdominal, vaginal and laparoscopic hysterectomy. At the beginning of the 20th century, surgery was radicalized in addition to hysterectomy, both adnexectomy (removal of the ovaries and fallopian tubes), collpectomy of upper and middle third vagina, lymphonodectomy of regional lymph nodes and ectomy parameters on both sides (Jensen et al., 2004). And today is current Wertheim-Meigs operation. The fact that metastases in regional lymph nodes occurs in a fairly high percentage at all stages of the disease and that the primary cervical lesion spreads to surrounding anatomical structures without clear clinical symptoms and signs make the surgical method inefficiently effective. Therefore, radiotherapy is used in the treatment of most of these patients, alone or in combination with surgical treatment (Jensen et al., 2004). On Figure 13 is shown radical hysterectomy for early stage of cervical cancer.

Today it is accepted in almost all oncology and radiology centers of the world that the combined cervical cancer therapy is the most effective. Intracavitary brachytherapy and teleradiotherapy are combined (Jesse and Aronowitz, 2015). The first way is destroying the malignant tissue on the cervix itself and its immediate surroundings, while others are destroying secondary deposits in the area of parametrium, regional and juxtaregional lymph nodes and other organs of the small pelvis. Intracavitary brachytherapy of cervical cancer has its own path of development (Jesse and Aronowitz, 2015). Radium-226 is applied to the area of transvaginal malignant lesions of the cervix. In some European centers, which dealing with the treatment of cervical cancer before the Second World War, special schools are also developed that a introduce a special method of intracavitary brachytherapy of cervical cancer. The most famous are the Stockholm, Paris and Manchester methods that are still applied in many centers. Thanks to the great progress in the production of artificial radioisotopes, the radium begins to be replaced by artificial radionuclides Co-60, Cs-137, Ir-192 and other gamma emitters (Turrel et al., 2005). In several of the most developed countries of the world (United States, Japan, Russia, France) for intracavitary brachytherapy of cervical cancer is used californium (Cf-252), which is a beta, gamma and neutron emitter (Banerjee and Kamrawa, 2014). Radioactive focuses are applied to the cervical channel, which is previously dilated into the vaginal vaults. An endocervical probe is inserted in length from 4 to 8 cm, depending on the depth of the uterine cavity and focuses on the area of vaginal fornix, usually two, whose supports are called ovoids. Radioactivity of the source per one complete application did not exceed 3-4 GBq (100 mCi). The radiation lasts for 1–2 days continuously and it is repeated one, two or more times with breaks between two consecutive applications from 1 to 2 weeks. Manual “afterloading” technique is used in large centers in which the incidence of cervical cancer is high (several hundred patients per year), where there was more irradiate of staff in application halls and departments in the use of classical intracavitary brachytherapy, since the time of transvaginal radiation lasted hours and days (Jesse and Aronowitz, 2015). To protect staff against ionizing radiation and improve irradiate patients at the beginning of the seventh decade of twentieth century, the new techniques are introduced into clinical practice. It is manual “afterloading” technique, which use special guide or carrier of focus (Jesse and Aronowitz, 2015). The carriers are first applied to the cervix area and subsequently the radioactive focus is taken manually from the plumbeous container and insert into these focus carriers. In this way the staff in the application halls is protected from ionizing radiation, as well as the staff that transported patients from the application halls to the department (Banerjee and Kamrawa, 2014). Henkel, a US radio therapist of German origin, introduced this technique to clinical practice. The system consists of three catheters made of stainless steel: one is applied, to the cervical channel and the uterine cavity and two with semi-spherical ovoids are applied at the top in the area of lateral vaginal fornix. Catheters are interconnected. After application, they are fixed by tamponing the vagina with sterile gauze (Banerjee and Kamrawa, 2014). Then, after applying this system in the application hall, the patient is taken to the department and while the patient is in bed, it is inserted C_0_-60 focuses of low intensity into the applied catheter (Ntekim et al., 2010). These focuses are taken manually from lead container into the uteral probe (short or long probe) 2^∗^0.059 GBq or 3^∗^0.059 GBq. At the end of 1976, a manual after loading technique with low intensity Cs-137 began to be applied at the Radiological Institute in Belgrade. Three plastic catheters are used, one is applied to the cervical channel and the uterine cavity and two with ovoids at the top in the area of the lateral vaginal vaults. Catheters are fixed with gauze tampons. Then, after applying this system in the application hall, the patient is taken to the department and while the patient is in bed, it is inserted Cs-137 focuses from the lead container and manually: long probe consisting from three focuses arranged in tandem (220+20+15 mCi) or two ovoids, each with 20 mCi. In the combination of long probe and two ovoids (total radioactivity was 95 mCi), the dose rate at point A was 1.41 cGy) (Jesse and Aronowitz, 2015). The radiation duration per application lasted from 33 to 39 h. Applications were performed twice with a break of 2 weeks (Jesse and Aronowitz, 2015). The dose at point A was 6500 cGy. In the mid-seventh decade of the 20th century, the so called “remote afterloading” technique with low, medium and high intensity sources. The principle is similar to manual technique, except that in this technique the radioactive focuses from the lead container is automatically inserted into the carriers in separate spaces, whose walls are sufficiently thick from the materials that completely protects staff from ionizing radiation (Jesse and Aronowitz, 2015).

On the principle of “remote afterloading” technique (the technique of subsequently filling at a distance) the machines: Katetron, Kiritron, Buhler, Brahitron, Toshiba, AGAT-V, Selektron HDR, Mikroselectron and others. One of the first machines, using “remote afterloading” technique and radionuclide Co-60 high radioactivity is Katetron (manufactured in England) (Ntekim et al., 2010). Focus guides represent three catheters made of stainless steel: one is applied to the cervical channel and the uterine cavity, two with ovoids at the top in the vaginal vaults. By the fixation system, the catheters are attached to the gynecological table, where the patients lie in the position for lithotomy and then the patients are transported to the bunker for radiation, where is placed a lead container with the high radioactivity focus Co-60 (Ntekim et al., 2010).

The patient remains alone in the bunker. On the dashboard in the adjacent room, the time of radiation is determined and then the focus automatically transmitted through semiflexion cables. in catheters that were previously in the application hall placed in the area of the malignant lesion. The radiation lasts only a few minutes. The focuses are then automatically returned to lead container and the patient is released from catheter and taken to the department or goes home (if outpatient radiated). Applications are administered once or twice a week in 3 to 6 times and doses in point A range from 500 to 1000 cGy per application. During the last few decades, “remote afterloading” technique has been improved. Selectron HDR (high efficiency sources Co-60) and microselectron (it is used a one source of Ir-192 high radiation and small size, which moves during radiation are machines that realize the requirements of clinical brachytherapy to the fullest extent (Ntekim et al., 2010; Jesse and Aronowitz, 2015). These machines are supplied with computer system by means of which leads to the volume distribution of radiation dose. Probably these technological solutions represent the ultimate range in brachytherapy (Jesse and Aronowitz, 2015). In transcutaneous radiation, the dose is calculated in the middle of the AP diameters in the pelvic region (Fernandes et al., 2012). These doses range from 40 to 60 Gy divided into 20 to 30 fractions for 4–6 weeks. Since transcutaneous radiation is combined with intracavitary and therefore used by central lead modifiers, the doses range from 80 to 90 Gy in point A and from 60 to 70 Gy in point B (intracavitary + transcutaneous). The total rectal dose should not exceed from 70 Gy, provided that is given transcutaneous 10 Gy per week (2 Gy per day) and intracavitary with low intensity sources, the length of treatment per application is 24–48 h (Follen et al., 2003). When metastases are present in the paraaortic lymph nodes, this area is radiated over two opposite parallel fields-front and back, which extend from the top of the first lumbar vertebra to the upper edge of the pelvic field (0.5 to 1 cm spacing). The width of these fields ranges about 8 cm, and the dose is catalyzed on the center of the AP diameter of the irradiated volume and amounts 45 Gy divided into 22–25 fractions (5 fractions per week). In terms of the order of transvaginal and intracavitary radiation, there are several variants: transcutaneously followed by intracavitary, intracavitary, transcutaneous, transcutaneous + intracavitary + transcutaneous or concomitantly transcutaneous and intracavitary radiation. Combined surgical-radiation cervical cancer treatment can be performed in three ways: radiation + surgery, surgery + radiation, radiation + surgery + radiation (Jesse and Aronowitz, 2015). Preoperative radiation can only be performed by transcutaneous route or by intracavitary radiation treatment is not excluded. Postoperative radiation cervical cancer treatment is performed only via transcutaneous or transvaginal pathway and often there are combined two methods (transcutaneous + intracavitary) usually 3–6 weeks after surgery. Also, good results are given by combination: intracavitary radiation of primary lesion + surgery + transcutaneous pelvic irradiation (in metastases in regional lymph nodes) (Fernandes et al., 2012). When radiotherapy is combined with surgery by rule the radiation dose is less than at application of radical radiation treatment. Efforts to prevent cervical cancer in women around the world are focused on screening with Papanicolaou (PAPA) tests and the treatment of precancerous lesions. Several methods and tests can be used as screening methods for cervical cancer: visual examination of the cervix by the naked eye using Lungol solution (Jesse and Aronowitz, 2015).

The safe setting of diagnosis requires quickly and energy therapy. At zero stage, the treatment is operative and the choice of the method depends on the age of the patient (woman) whether she gave birth or she wants more children (Ntekim et al., 2010). Young women and women who want to give birth should preserve the uterus and its functions and remove the malignant hearthstone, which is achieved by removing the cervix tissue surrounding the endocervix until its inner mouth. The removed part of the cervix is directed to a pathohistologist, who checks the diagnosis of preinvasive carcinoma at serial cross-section. Operational therapy performed in this way does not disturb the generative function of woman, so women are able to later become pregnant, bear pregnancy and give birth with regular and caring control of the gynecologist (Jesse and Aronowitz, 2015).

A conservative surgery-conization, if it is established with certainty that it is a preinvasive carcinoma is also performed in pregnant women, who with regular control bring pregnancy to the end and give birth. If the diagnosis of preinvasive carcinoma of the cervix is securely set, older elderly women, who do not want to give birth can perform total hysterectomy (Jesse and Aronowitz, 2015).

If, according to a clinical finding, it is assumed that behind of a zero stage can suppress invasive carcinoma, then it is better for the older women to do the conization or amputation. Based on the histological finding from the obtained tissue it should be decided whether this operation is sufficient, which is the case at a negative finding, or the treatment must continue with radical hysterectomy and radiation, if an invasive form of a malignant lesion is detected during the histological examination (Jesse and Aronowitz, 2015).

Opinions about the treatment of invasive forms of cervical carcinoma have been divided. Some gynecologists recommended the operative therapy of the initial clinical forms of cervical cancer and others are supporters of radiation. Most often, the first and the initial second stage is treated operatively, if there are no other contraindications for surgery (excessive obesity, heart disease, expanded veins, kidney disease, etc.). Therefore, an extensive abdominal hysterectomy is performed with the removal of adnexa, parametrium, regional lymph nodes and upper part of vagina (Jensen et al., 2004).

Before and after hysterectomy, percutaneous or intracavitary ionizing radiation is recommended. Patients in the late second or third stage do not undergo surgery, but they are only treated with combined ionizing radiation. Certain operators in these, but selected cases in some countries (United States, Japan, etc.) still perform the operational exertation of the whole small pelvic by the Brunswick method. The operational method is not universally accepted. Patients in the fourth stage are not treated causally, but their medicament and especially opiates are relieved their last days of life. The prognosis of cervical cancer depends on the stage in which the treatment is performed. When it comes to preinvasive forms of the disease, healing is achieved in all patients (Jesse and Aronowitz, 2015). With the advancement of the malignant invasion process, chances for healing are reduced. Thus, e.g., the percentage of survivors 5 years after the performed treatment in the first stage is about 80%, then in the second stage about 50% and in the third stage it is very small.

Therefore, the most effective measure in treating cervical cancer is an early diagnosis. Radiotherapy, alone or in combination with surgery is the method of choice in the treatment of this malignancy. The success in treating of this tumor is estimated on the basis of a five-year survival of treated patients without a local recurrence.

## The Role of Bile Acids in Treatment of Various Cancer *In Vivo* and *In Vitro* Models

Numerous epidemiological studies have confirmed the association of bile acids and development of colon cancer. In patients with colorectal adenomas and carcinomas are elevated concentrations of deoxycholic acid and lithocholic acid, as secondary bile acids, in systemic circulation and in the lumen of the colon (Barrasa et al., 2013). Bearing in mind the association of high levels of bile acids in the lumen of the intestine and the development of colon cancer, the consequences of prolonged exposure of intestinal mucosa with bile acids were intensively examined (Brestoff and Artis, 2013). It was found that hydrophobicity is the main determinant of toxicity of bile acids and depends from the number position and orientation of hydroxyl groups, as well as from conjugation with glycine or taurine (Van Heumen et al., 2012). It is also known that cholecystectomy, which leads to an increase exposure to intestine by bile acids, a predisposing factor for colorectal carcinoma (Lagergren et al., 2001). Ursodeoxycholic acid is the most hydrophobic bile acids, while deoxycholic acid and lithocholic acid are the least polar and therefore the most toxic bile acids, which can contribute to the development of colorectal cancer (Sun and Kato, 2016). The pathogenesis of colorectal cancer is a long-lasting progressive process, which involves gradual accumulation of genetic and epigenetic alterations, often accompanied by intestinal imbalance microflora, which leads to the transformation of the normal mucosa of the colon in invasive carcinoma (Barrasa et al., 2011). The specificity of the development of colon carcinoma is a consequence of the characteristic morphology of the intestinum (Degirolamo et al., 2011). Intestinal epithelium represents the first line of defense against pathogens and other harmful factors and exhibits a range of physiological functions, primarily digestive and absorbent. Several signaling pathways are of key importance in the regulation of intestinal homeostasis of which the most significant is the Wnt/β-catenin signal path. The bile acids may modulate different processes of relevance to the development of colon cancer. It has been found that bile acids can to influence the process of apoptosis of intestinal cells, to promote the proliferation of the epithelium of the colon, modulate inflammatory pathways in the cell, initiate oxidative damage to the DNA or lead to disorders of mitotic activity of cells (Gadaleta et al., 2017). In addition, the interaction of bile acids and intestinal microbiomes can significantly alter the function of the gastrointestinal tract and participate in pathogenesis of colon carcinoma (Brestoff and Artis, 2013; Gadaleta et al., 2017). Different bile acids are not equally involved in apoptosis, cellular processes proliferation and cancer development. Apoptosis and proliferation are very related processes and in fact, the balance between these two cellular processes determines the tissue homeostasis. Ability of induction of apoptosis depends directly on hydrophobicity, but also on the concentration of bile acids. While hydrophobic bile acids, such as deoxycholic acid and chenodeoxycholic acid are well known in apoptosis inducers of cell damage, hydrophilic ursodeoxycholic acid has a cytoprotective effect, which is why it is used in therapy cholestatic diseases of the liver. Under physiological conditions, the concentrations of bile acids in the colon are much lower than those in the gallbladder (∼ 300 mM) or in the small intestine (∼10 mM), but people with high intake of fat-rich foods, they can reach a value of about 1 mM, of which about 700–800 μM makes very cytotoxic deoxycholic acid (Barrasa et al., 2013). Two different mechanisms have been described in which hydrophobic bile acids achieve their own cytotoxic effect. High concentrations of bile acids damage cellularity membrane due to their detergent properties and lead to cell death, most often necrosis (Barrasa et al., 2013). In lower concentrations, bile acids can induce cell death and specifically, via the receptor. More frequent is the internal pathway of induction of apoptosis, because of the fact that hydrophobic bile acids easily enter in the cell’s by passive diffusion and lead to the generation of ROS. The oxidative stress of the cell induces the formation of pore in the mitochondrial membrane, the release cytochrome c and further reactions of caspases that lead, finally, to apoptosis of the cell. Alternatively bile acids can induce apoptosis by externally pathway via CD95/Fas and TRAIL-R2 receptors. Hydrophobic bile acids, such as deoxycholic acid, can also be in lower concentrations to effect on the cell membrane by inducing cholesterol aggregation and phosphorylation of caveolin-1, which leads to aberrant signaling of EGFR, inducing further activation of MAPK signaling pathways, as well as NF-κB and caspase-8 (Solimando et al., 2011). It was also established that bile acids can lead to the release of calcium from the endoplasmic reticulum (ER), increase in calpain and caspase-12 activity, respectively, to induce apoptosis in hepatocytes through stress ER. Hydrophilic bile acids, of which the most important is ursodeoxycholic acid can induce signal pathways to survive the cell and thus to achieve their cytoprotective effect. These signal paths have been developed to protect the cells from pathological apoptosis and include NF-κB, PI3K and MAPK signaling pathways. Stimulus for survival induces these intracellular signal pathways mainly through activation transmembrane receptors. Ursodeoxycholic acid realizes its cytoprotective and antiapoptotic effects by preventing the formation of ROS and translocation of proapoptotic protein BAX from the cytosol to mitochondrial membrane (Amaral et al., 2009). It has been established that long-term exposure of enterocytes to sublethal concentrations of bile acids, leads to resistance to apoptosis, which further contributes to carcinogenesis in the colon (Bernstein et al., 1999). Also, hyperproliferative effects of deoxycholic acid on various cell lines of colon carcinoma have been demonstrated (Moschetta et al., 2003). Significant effects of increased concentrations of hydrophobic bile acids in lumen of intestinum is also seen in the induction of proliferation of the epithelium of the colon mediated activation of EGFR and downstream activation of protein kinase C (PKC). Perturbations on level of cell membranes mediated by exposure of the epithelial cells of the colon to high concentrations deoxycholic acid, are the result of a combination of various factors, including reduced fluid flow, cholesterol distribution in the microdomains of the membrane, as well as chemical properties of bile acids. In addition to activating NF-kB, deoxycholic acid in epithelial cells of the colon leads to activation of AP-1 as a result of PKC stimulation. It has been established that ursodeoxycholic acid has the opposite effect in relation to deoxycholic acid (Degirolamo et al., 2011). The bile acids are also included in other signaling pathways that regulate the cell cycle and contribute to the development or regression of malignancy. The effect of bile acids is significant for the process of inflammation, primarily the effect on the NF-κB signal pathway, as well as the modulation of metabolism prostaglandin (Miyaki et al., 2009). Numerous studies have shown an elevated level of prostaglandin E2 (PgE2) in colorectal adenomas and carcinomas, indicating the important role of this compound in colon carcinogenesis. It was first shown that hydrophobic bile acids, deoxycholic acid and chenodeoxycholic acid can induce expression of the COX-2 gene, stimulating the synthesis of prostaglandins (Tucker et al., 2004; Van Heumen et al., 2012). For unconjugated chenodeoxycholic acid is also *in vitro* on the cell line of human colorectal cancer HT-29 has been shown to lead to a strong suppression of gene transcription for a 15-hydroxyprostaglandin dehydrogenase (PGDH), a key enzyme in catabolism of prostaglandin (Miyaki et al., 2009). PGDH expression has been significantly reduced in a number of tumors, including colorectal carcinoma, and the mechanism involved in these processes is the activation of the PKC axis – ERK1/2 – Egr-1-Snail, and finally PGDH (Sun and Kato, 2016). The above data indicates a direct connection of elevated levels of hydrophobic bile acid (chenodeoxycholic acid) and carcinogenesis in gastrointestinum (Khare et al., 2008). Ursodeoxycholic acid, on the other hand, alleviates inflammatory processes, primarily through the suppression of COX-2 expression, as demonstrated *in vitro* on HT-29 cells, as well as on the azoxymethane experimental model of colon carcinoma in rats (Khare et al., 2008). Hydrophobic bile acids lead to the generation of ROS in the cells and, except that they can induce the internal pathway of apoptosis, can lead to disorders in mitotic activity cell. Bile acids can induce genomic and chromosomal instability in the epithelial colon cells, which are characteristic of colorectal cancer, through several mechanisms. It has been shown that bile acids can lead to aneuploidy, that is prevents the separation of chromosomes during mitosis (Payne et al., 2010). They can also lead to oxidative DNA damage and stopping of the cell cycle in the G1 and/or G2 stages, but also to the irregular placement of chromosomes into the equatorial plane of the cell during metaphase and to the occurrence multipolar division due to the aberrant number of centriols (Payne et al., 2010).

Bile acids are present at high concentrations in breast cysts and in the plasma of postmenopausal women with breast cancer. The farnesoid X receptor (FXR) is a member of the nuclear receptor superfamily that regulates bile acid homeostasis. FXR was detected in normal and tumor breast tissue, with a high level of expression in ductal epithelial cells of normal breast and infiltrating ductal carcinoma cells (Bishop-Bailey et al., 2004). FXR was also present in the human breast carcinoma cells, MCF-7 and MDA-MB-468. Activation of FXR by high concentrations of ligands induced MCF-7 and MDA-MB-468 apoptosis (Otte et al., 2003). At lower concentrations that had no direct effect on viability, the FXR agonist GW4064 induced expression of mRNA for the FXR target genes, small heterodimer partner (SHP), intestinal bile acid binding protein, and multidrug resistance-associated protein 2 (MRP-2), and repressed the expression of the SHP target gene aromatase. In contrast to MRP-2, mRNA for the breast cancer target genes MDR-3, MRP-1, and solute carrier transporter 7A5 were decreased. Although multidrug resistance transporters were regulated and are known FXR target genes, GW4064 had no effect on the cell death induced by the anticancer drug paclitaxel (Costarelli and Sanders, 2001). Breast cancer is the most common form of cancer in women in the Western world. Its incidence is epidemiologically linked to high-fat diets, which increase the amounts of bile acids in the body (Cho et al., 2003). High levels of the plasma bile acid deoxycholic acid (DCA) are found in postmenopausal women newly diagnosed with breast cancer, whereas chenodeoxycholic acid (CDCA) is present at high Amol/L levels in breast cyst fluid (Javitt et al., 1994; Costarelli and Sanders, 2002). Moreover, CDCA can inhibit the growth of the breast carcinoma cell line MCF-7, whereas glycol-CDCA causes proliferation. In breast cancer cell lines, FXR activation down-regulates the breast cancer target genes; local estrogen producer aromatase and the transporters MDR3, MRP-1, solute carrier transporter 7A5 (SLC7A5); and inhibits cell proliferation. It also induces the expression of the known FXR target genes SHP, IBABP, and MRP2 (Baker et al., 1992).

Bile acids act as promoters, but not as carcinogens in people. Given the cytotoxicity of bile acids and the impact on the development of cancer, strict control of their intracellular and systemic serum concentrations below normal conditions are achieved by the network of pathways of the nuclear receptors to which they are attached. Recently studies have found that the expression of nucleic receptors that are associated with bile acids, primarily FXR, decreased in colon carcinoma cells. Therefore, they are current therapeutic strategies aimed at reactivating FXR in colon carcinoma cells (Modica et al., 2014). In physiological conditions, FXR is expressed exclusively in fully differentiated intestinal cells epithels, primarily ileum and column, and with the reduction of the degree of differentiation, the expression of this nuclear receptor. It has been established that CDX2 is a transcription factor, which regulates the differentiation of intestinal cells in the organism, necessary for adequate expression FXR in the intestinum. A link was also found between the loss of expression of FXR and carcinogenesis in the liver and intestinum (Modica et al., 2008). The level of expression of FXR is in a negative correlation with degree of malignancy, and in a positive correlation with favorable clinical outcome. Experiments with FXR-deficient mice showed that the lack of FXR worsens both hepatic and intestinal inflammation, as well as promote carcinogenesis in gastrointestinum (Bailey et al., 2014). While lowering FXR levels in the cells contributes to the development of tumors, the activation of this receptor in colon carcinoma cells leads to the suppression of proliferation of these cells and induction of apoptosis. The loss of FXR function in mice has the effect increased susceptibility to chemically induced carcinogenesis in the colon, while in transgene mice with constituent activity of FXR in the intestinum reduced the risk of development colorectal cancer, and tumor growth itself. It has recently been established that it is inactivation of the APC gene, which is present in most types of colon carcinoma, monitored reduced expression of FXR, and that this is most likely the result of the methylation of promoter FXR gene, which further prevents transactivation of the target gene of the FXR receptor and leads to an increase expression of COX-2 gene expression. The methylation of the FXR gene promoter was also determined in different ways cell lines of colon carcinoma, as well as in clinical samples of colon tumors (Maran et al., 2009). And other receptors which bind for bile acids have a role in the carcinogenesis carcinogenesis process. It is known that vitamin D and its analogs have a protective effect on initiation and tumor progression, as confirmed in various *in vivo* animal carcinogenesis models. *In vitro* and *in vivo* studies, antiproliferative effects of vitamin D have been demonstrated, and have been identified numerous target genes, such as the regulators of the cell cycle p21 and p27 and the apoptosis regulators BAX and BCL-2 (Pan et al., 2010). PXR is known to play a role in cancerogenesis (Zhou et al., 2008; Ouyang et al., 2010). It has been shown to be by activating PXR, HCT-116 cell carcinoma cells protect against apoptosis induced by deoxycholic acid and cytotoxic drug doxorubicin, through molecular mechanisms involving induction anti-apoptotic gene BIRC2 and MCL1, as well as inhibition of proapoptotic gene BAK1 and TP53. Although a large number of studies indicate the PXR receptor as an antimicrogenic factor, it has been established that the transfection of PXR into the HT-29 colon carcinoma cells results in suppression of proliferation, as well as reduced tumor growth in the HT-29 xenograft model in mice (Selmin et al., 2016).

This is accomplished through controlling the cell cycle by influencing p21 and a signal pathway involving the transcription factor E2F and tumor suppressor Rb. Bile acids can participate in pathogenesis of colon carcinoma and through interaction with intestinal microbiome (Sun and Kato, 2016). The mice are raised in sterile conditions or they are treated with antibiotics, have lower concentration of toxic, secondary bile acids in feces and more systemic concentrations of conjugated bile acids compared to mice with preserved intestinal microflora. The relationship between microbiome and development of colorectal cancer has been established (Sun and Kato, 2016). It is known that malignant transformation is most common in distal areas of intestinum, where there is the highest number of intestinal bacteria. Carcinogenesis in the experimental animals depends on the presence of microorganisms in the lumen of the gut. Although a great effort has been made by scientists to identify the individual microorganism that is the cause of carcinogenesis in the column, such as *Helicobacter pylori* in the stomach. It has been proven that colon cancer is associated with a change in microbial in terms of increase and decrease certain bacterial species, as well as the change in the level of certain bacterial metabolites (Brestoff and Artis, 2013).

Thus, for example, nitrogen compounds as metabolic products of intestinal bacteria, consistently elevated in colon carcinoma, while the level of short-chain fatty acids, first of all butirata, most often reduced. Changed composition of intestinal microflora and their metabolites creates a microclimate that promotes inflammation, proliferation, and neoplastic transformation. The relationship of excessive fat intake with colorectal development has been established cancer, which is explained primarily by the increased secretion of bile and high concentrations of secondary bile acids in the colon. The change of diet from low to a high fat intake in rats results in a change in the composition of the intestinal microflora in terms of increasing relationship of the type of bacteria Firmicutes to the type Bacteriodetes, that is predisposing factor for the development of colonic inflammation and consequent carcinogenesis (Taira et al., 2015).

## Bile Acids as Therapeutic Agents in Treatment of Cervical Cancer

The synthetic derivatives of ursodeoxycholic acid and chenodeoxycholic acid such as HS-1183, HS-1199, and HS-1200 are used in treatment of cervical cancer. Furthermore, the increase in p21 WAF1/CIP1 by synthetic bile acids was strongly associated with proliferating cell nuclear antigen (PCNA), which is required for the process of DNA synthesis by DNA polymerases. Natural products in treatment of cervical cancer are suitable alternatives that can be used instead of platinum-based drugs in control of cervical cancer, which show some harmful side effects. HS-1199, HS-1200, and HS-1183 (the synthetic derivatives of ursodeoxycholic acid) induced apoptosis in SiHa cells in a dose-dependent manner and this effect was associated with the activation of transcription factors AP-1 and NF-kB (Eunok et al., 2005). The activation of the JNK/AP-1-signaling pathway plays an important role in SiHa cell death.

It is very important that the synthetic derivatives of chenodeoxycholic acid and ursodeoxycholic acid are capable of inhibiting cell proliferation and inducing apoptosis in SiHa cells.

Eunok et al. (2005) evaluated the effects of synthetic derivatives of ursodeoxycholic acid and chenodeoxycholic acid on the cell line of cervical cancer and suggested that apoptosis was caused by NF-kB regulation of apoptotic gene such as Bax. HS-1183 is conjugate of ursodeoxycholic acid (UDCA) with *N*- [(3α,5β,7β)-3,7-dihydroxy-24-oxocholan-24-yl] L-phenylalanine benzyl ester, HS-1199 is conjugate of chenodeoxycholic acid with (*N*-[(3α, 5β, 7α)-3,7-dihydroxy-24-oxocholan-24-yl] L-phenylalanine benzyl ester and HS-1200 is a conjugate form of chenodeoxycholic acid with (*N*-[(3α, 5β, 7α) -3,7-dihydroxy-24-oxocholan-24-yl] β-alanine benzyl ester. Ursodeoxycholic acid has the ability to inhibit the dynamic and multiple functional p53 (Hietanen et al., 2000). The suppressor protein of tumor, p53 is crucial for the elimination of damaged cells from the replicating pool to protect the body from malignant transformation. Engagement of the p53 signal pathway occurs as a response to a wide range of stressors, substantially and extrinsic in the cell, which stabilize and affect on the p53 series of post-translocation modifications. Ursodeoxycholic acid inhibits p53 induction and stabilization through an independent caspase mechanism (Hietanen et al., 2000). Most importantly, bile acid inhibition of p53 induced apoptosis was associated with reduced p53 DNA binding activity (Hietanen et al., 2000). Subcellular localization of induced apoptosis p53 was also altered by the influence of ursodeoxycholic acid, p53 and pro-apoptotic Bax hepatocytes are regulated by deoxycholic acid. CDCA derivatives, HS-1199 and HS-1200, showed stronger activity than an UDCA derivative (HS-1183) in treatment of cervical carcinoma (Eunok et al., 2005). This synthetic bile acid derivatives activate JNK/AP-1-signaling pathway, which plays an important role in human cervical cancer cell death (Eunok et al., 2005). In human cervical carcinoma cells, p53 was disrupted by HPV E6 oncogene. There is a significant increase of Bax expression without Bcl-2 expression in human cervical carcinoma cells treated with the synthetic bile acid derivatives (Eunok et al., 2005). Ratio of Bax and Bcl-2 is increased and might contribute to the initiation of apoptosis in the synthetic bile acid derivatives-treated cells (Eunok et al., 2005).

Prolonged activation of AP-1 and NF-kB may play an important role in determining the cell death in response to oxidative stress, anti-cancer drug and growth factor withdrawal (Eunok et al., 2005).

The synthetic bile acids induce apoptosis in a JNK dependent manner in SiHa human cervical cancer cells, via induction of Bax (Eunok et al., 2005). The activation of JNK/AP-1 signaling pathway was involved in the synthetic bile acid derivatives-triggered apoptosis. CDCA derivatives, HS-1199 and HS-1200, showed stronger activity than an UDCA derivative (HS-1183) highly increased the expression level of c-Jun in SiHa human cervical cancer cells (Eunok et al., 2005).

Ursodeoxycholic acid reduces the number of Bax-positive cells, whose increase is induced by deoxycholic acid (Eunok et al., 2005). Transcriptional UDCA inhibition of DCA-induced hepatocyte apoptosis is regulated by expression of the p53/Bax signal molecule. Ursodeoxycholic acid suppresses NF-kB through functional modulation of glucocorticoid receptor and via NF-kappa B-dependent transcription (Eunok et al., 2005). Synthetic derivative of ursodeoxycholic acid HS-1183 induces apoptosis in human cervical carcinoma cells. Polyamine 3’-Azido-3’-deoxythymidine (AZT) was conjugated with bile acids, wherein the conjugates were tested by preliminary assays on HeLa cells of human cervical carcinoma *in vitro* by the standard MTT method (Eunok et al., 2005; Dimao et al., 2007). Conjugates based on dipropylene-triamine and stearyl alcohol have significant anticancer activity against the HeLa cell line compared to AZT, which contribute low inhibition of HeLa cell line of cervical carcinoma. The conjugation between bile acid-polyamine amide and AZT definitely improved the antitumor activity of AZT and its H-phosphate. A series of new bile acid polyamine-AZT phosphoramidate conjugates significantly suppressed the growth of HeLa cervical cancer cells (Dimao et al., 2007).

The therapeutic potential of bile acids, that build a complex with metals has attracted the great interest of scientist. Metals exhibit unique characteristics, such as redox activity, variable mode of coordination and reactivity to the organic substrate (Pérez-Andrés et al., 2008). These properties become an attractive test in the design of metal complexes, which selectively bind to the target biomolecule (bile acids) with the resulting change in the cell proliferation mechanism. Platinum compound, especially cisplatin are used as catalysts in cervical cancer therapy (Pérez-Andrés et al., 2008). Clinical use of platinum complexes for which the bile acids bind is based on the desire to achieve the death of tumor cells and the spectrum of candidate drug activity in the treatment of cervical cancer. Cervical squamous intraepithelial lesions and invasive cervical squamous cell carcinoma were immunostained for Bcl-2 and Bax proteins and compared with benign cervical squamous epithelium to assess the involvement of Bcl-2 and Bax proteins in cervical carcinogenesis from the intraepithelial neoplastic stage to overtly invasive carcinoma.

The cholic acid nanoparticle system is functionalized in a star-shaped block copolymer consisting of PLGA and vitamin E D-alpha-tocopheryl polyethylene glycol succinate (TPGS) for the sustained and controlled delivery of docetaxel to treat cervical cancer, which TPGS with three demonstrated superior performance *in vitro* and *in vivo* (Mu and Feng, 2003; Lapienis, 2009). Cholic acid is a superior carrier of docetaxel and it is used as a model of an anticancer drug, which in its effective encapsulation in the treatment of cervix cancer is used a block copolymer in the form of a CA-PLGA-b-TPGs star (Xiaowei et al., 2013). Poly(lactide-co-glycolide) (PLGA) is one of the most widely investigated biodegradable polymers for biomedical applications (Ouyang et al., 2012).

**FIGURE 14:**
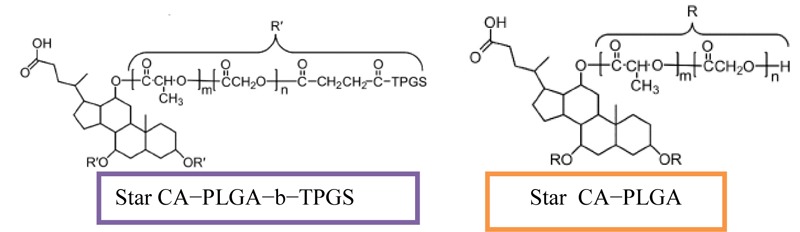
Structure of Star CA-PLGA-b-TPGS and Star CA-PLGA.

On Figure 14 is shown block copolymer Star Cholic acid-polylactic-Co-glycolic acid D-alpha-tocopheryl polyethylene glycol succinate and star Cholic acid-polylactic-Co-glycolic acid.

Platinum compounds, particularly cisplatin, which bind with bile acids are the heartbeat of the metal-based compounds in cancer therapy. Clinical use of platinum complexes as an adjuvant in cancer therapy is very important and it is based on the desire to achieve tumor cell death and the spectrum of activity of the candidate drug. Such complexes are used in treatment of cervical carcinoma (Pérez-Andrés et al., 2008). The importance and therapeutical potential of metal complexes in therapy of cervical cancer-reflected in their redox activity, variable coordination modes and reactivity toward the bile acids. These properties are very important in the design of metal complexes that selectively of proliferation. Satraplatin, bis-(acetate)-amine dichloro-(cyclohexylamine) platinum (IV), is the first orally bioavailable platinum drug in cervical carcinoma (Pérez-Andrés et al., 2008).

Bisursodeoxycholate (ethylenediamine) platinum (II) [Pt(UDC)_2_(en)] is characterized by important cytotoxicity against HeLa cervical carcinoma cells and this effect already being clearly detectable after 24 h. The cytotoxicity of tumor cells achieved by [Pt(UDC)_2_(en)] complex against S+G_2_/M- as well as G_0_G_1_-phase tumor cells and not only against cycling HeLa cells as found as cisplatin.

This citotoxicity seems to overcome resistance to cisplatin owing to the use of different mechanisms of action by bile acids and cis DDP (Pérez-Andrés et al., 2008).

**FIGURE 15:**
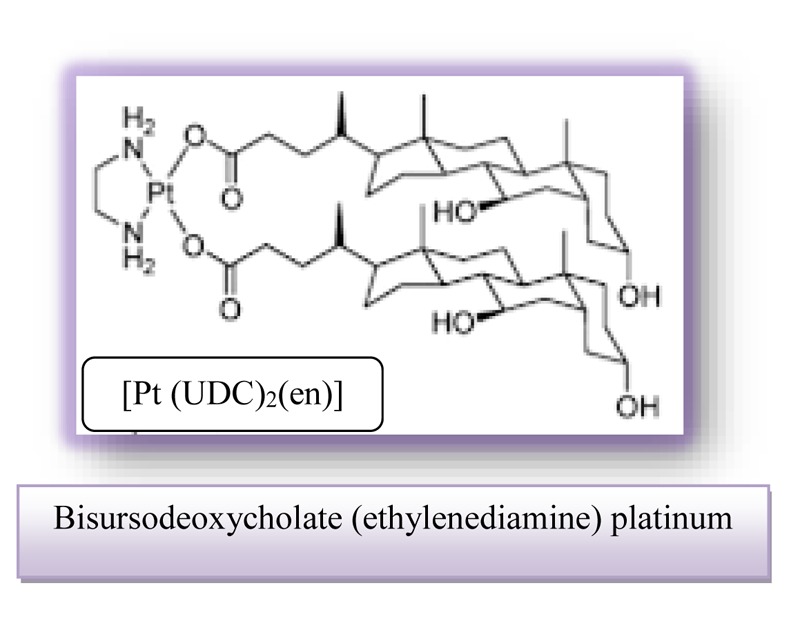
Structure of Bisursodeoxycholate (ethylenediamine) platinum.

On Figure 15 is shown structure of Bisursodeoxycholate (ethylenediamine) platinum.

Cisplatin induced an accumulation of surviving cells at the G0/G1 cell cycle phases. No major changes were observed in the cell cycle distribution of HeLa cancer cells cultured with [Pt(en)(UDC)_2_] versus untreated cells. As noted for [Pt(en)(UDC)Cl], these observations indicate that [Pt(en)(UDC)_2_] exerts cytotoxicity upon resting cells as well as cycling cells.

Zn-UDCA significantly decreased the viability and proliferation of human tumor HeLa cell lines; applied independently, ursodeoxycholic acid expressed lower cytotoxic/cytostatic activity as compared to metal complexes and the sensitivity of the non-tumor embryonic Lep-3 cells to the effects of ursodeoxycholic acid and its metal complexes was compared or even higher than those of the human tumor cells (Pérez-Andrés et al., 2008).

Hela was the first cultured cancer cell line, which was derived from cervical cancer cells taken from Henrietta Lacks in 1951. Since then, several cervical cancer cell lines including SiHa, CaSki, C-33A, and ME-180 were established. It is worth to mention that these cell lines do not have equal value as tumor models, thus the *in vitro* results of drug efficacy experiments are different. Cancer cell lines in three-dimensional (3D) culture models indicate higher malignancy, invasive characteristic, and drug resistance than classic two-dimensional (2D) cell culture (Pérez-Andrés et al., 2008).

HeLa, SiHa, CaSki, C-33A, and ME-180 human cervical cancer cells are destroyed with Ni-UDCA, Cu-UDCA and Zn-UDCA complexes during the treatment of cervical cancer and these complexes show much better effects compared to ursodeoxycholic acid itself (Pérez-Andrés et al., 2008).

## Discussion

An important role in the development of cervical cancer has HPV. Factors that increase the risk of developing cervical cancer are sexual activity at an early age, a positive history of sexually transmitted diseases, multiparity, smoking and lower educational level. Also, increasing the risk of cervical cancer associated with the length of use of oral contraceptives.

The prevalence of HPV infection in women without pathological changes in the cervix in the world is 11-12% and in women with H SIL and invasive carcinoma, it is about 90%. The prevalence of HPV in the population of women with normal cytology in Serbia is 11% and prevalence of HPV 16 or HPV 18 is in the range of 4.1% in women in normal cytology, 24.6% in women with L-SIL changes, 47.2% with H-SIL to 65.6% in cervical cancer patients. Women infected with HIV 1 are more susceptible to the development of HPV infection and SIL changes on the cervix. HPV 16 is most present in HIV positive women and H-SIL changes, while other HPV types are most present in the general population of women with L-SIL type changes. The prevalence of HPV infection in the HIV positive women is higher than in general populations. The risk of progression in H-SIL lesions and growth of cervical cancer is also higher. A less prevalence of cervical abnormality in women with HIV negative infection has been identified compared to HIV infection in women.

A long latent period between the initial exposure to HPV and the development of cervical cancer, the high prevalence of HPV infection in asymptomatic women and the fact that the malignant alteration process occurs only in one of several thousands of infected cells indicates that, although necessary, HPV infection is not sufficient for the development of cervical cancer. Frequently the presence of HPV in the younger age, which largery ends without health consequences suggests the existence of other necessary factors for the development of cervical cancer, such as the role of the immune system and genetic factors. Risk of cervical adenocarcinoma in HPV positive women has been statistically significantly increased in women with lower levels of education, with early beginning of sexual activity, with more sex partners, with a positive history of sexually transmitted diseases, with partners who have a history of sexually transmitted diseases. The risk of adenocarcinoma increases with the duration of hormonal contraception and with the number of pregnancies. In order to confirm the influence of other risk factors for cervical cancer, it is necessary that future studies include different ethnic populations with a large sample of respondents with a combination of genetic factors, age of respondents, parity, smoking and alcohol consumption. Women with chlamydial infection of cervical cancer have twice higher risk of developing cervical dysplasia compared to women who have not infection. Chlamydia trachomatis (CT) infection is an significantly risk factor for the development of cervical cancer in women. A Chlamydial infection is often associated with cytological atypia and dysplasia of the cervical epithelium.

Exposure to infection with Chlamydia trachomatis increases the incidence for persistent of HPV infection. This effect may be associated with an increased risk of cervical cancer. Chlamydia trachomatis infection may be predisposing factor for later HPV infection or vice versa, due to a similar way of sexually transmitted infection. HPV is the major etiological factor in the development of cervical cancer and bacterial vaginosis (BV) and other sexually transmitted infections increase the risk of cervical cancer. In addition to the role of viral factors in malignant transformation of the cell, host genetic factors also play a significant role. Human leukocyte antigen (HLA) genotype variations determine the immune response to HPV infection and thus its outcome.

Polymorphism p53 is often found in high level preinvasive lesions (severe dysplasia, H-SIL) and invasive cervical cancer In variations of type dysplasia of a mild degree L-SIL, the DNA remains in a circular form and does not integrate in in the host genome as opposed to the change of type H-SIL, where the DNA is integrated into the host genome at sensitive chromosome points.

Highly oncogenic HPV types 16 and 18 have genes E6 and E7, which produce proteins that can block the effects of p53 and Rb and thereby disrupt normal cell division, whereby the infected cells are divided without any control. The extreme growth of such cells leads to permanent changes in their genetic structure, the possibility of reparation does not exist and finally such cells are transformed into malignant. A premalignant disease can catch and break through the basal membrane, when it comes to invasion. Among the most important screening methods in the detection of premalignant and early malignancies cervical changes include exfoliative cytology (Papanicolaou test) and colposcopy, because they fully correspond requirements that each must fulfill screening method so that it can be applied in massive detection. These two methods, complementarily used are reliable in 95% cases and together with the cervical biopsy represent a triad that is sufficient to diagnose with certainty. Papanicolaou test and colposcopy should be routine methods in every gynecological ambulances and apply to each woman for a certain period of time. These methods allow the detection of premalignant conditions and the “zero” stage of cervical cancer, which is 100% curable and a microinvasive cervical cancer, which is asymptomatic and nearly 100% curable.

The latest discovery of mechanisms of oncogenesis and the role of E6 and E7 HPV proteins open up new possibilities for diagnosis, monitoring and prognosis of precancerous cervical lesions. All preventive and diagnostic-therapeutic measures and activities related to the prevention and polyamine early detection of cervical cancer, treatment and rehabilitation are carried out at all levels of health care. Countries with a well-organized health care system and long tradition of organizing preventive programs have significantly lower rates of incidence of cervical cancer. Primary prevention means activities aimed at improving and preserving health and preventing the onset of disease through reducing exposure to risk factors or through positive behaviors. It is estimated that primary prevention could prevent the emergence of two thirds of all cases of malignant diseases. Vaccine promoters believe that HPV vaccination could prevent up to 70% of squamocellular carcinoma and up to 85% of cervical adenocarcinoma, despite the fact that clinical studies have not shown that vaccines have prevented the development of cervical cancer. Linking the infectious agent with the development of intraepithelial neoplasia and cervical cancer cirrhosis, as well as the classification of HPV into oncology agent, made further progress in the attempt to eradicate HPV infection. Vaccination present a newer possibility of primary protection against viruses, whose infection leads to cytological abnormalities and the development of cervical cancer. The vaccine generated immunity specifically against HPV type 18 as the main causative agent of cervical cancer that is harder to detect by screening can reduce the development of adenocarcinoma for more than 90%, as well as 80 to 90% of all cases of cervical cancer in the world.

In order to find the most optimal and most effective screening, many studies have examined the efficiency of screening method individually or in combination of several methods.

Application of screening is the most important measure of prevention of cervical cancer, early detection and more successful treatment, significantly improves the quality of women life, preserves fertility and reduces costs of treatment from the economic point of view.

The synthetic derivatives of ursodeoxycholic acid (UDCA) and chenodeoxycholic acid (CDCA) are capable for inhibiting of cell proliferation and inducing apoptosis in SiHa human cervical carcinoma cells. The synthetic CDCA derivatives, such as HS-1199 and HS-1200 have a stronger growth inhibitory effect on cancer cells than an UDCA derivative, HS-1183 and synthetic bile acid derivatives induced apoptosis through activation of transcription factors AP-1 and NF-kB. The system of the cholic acid functionalized star-shaped PLGA-b-TPGS (CA-PLGA-b-TPGS), polymeric nanoparticles NPs control delivery of drug, such as docetaxel for treatment of cervical cancer. The conjugation achieved between the bile acid-polyamine amid and AZT definitely improved the antitumor activity of AZT and its H-phosphate 4, the compounds of dipropylenetriamine replaced by diethylenetriamine decreased the IC50 values of conjugates.

## Author Contributions

Both authors constructed an idea or hypothesis for research and/or whole manuscript, organized and supervised the course of the project or the article, performed logical interpretation and presentation of the results, provided personnel, environmental and financial support and tools and instruments that are vital for the project, contributed to taking responsibility in necessary function of literature review, and reviewed the article before submission not only for spelling and grammar but also for its intellectual content.

## Conflict of Interest Statement

The authors declare that the research was conducted in the absence of any commercial or financial relationships that could be construed as a potential conflict of interest.
